# Compromised CD8+ T cell immunity in the aged brain increases severity of neurotropic coronavirus infection and postinfectious cognitive impairment

**DOI:** 10.1111/acel.14409

**Published:** 2024-11-17

**Authors:** Katie L. Reagin, Rae‐Ling Lee, Luke A. Williams, Loren Cocciolone, Kristen E. Funk

**Affiliations:** ^1^ Department of Biological Sciences University of North Carolina at Charlotte Charlotte North Carolina USA

**Keywords:** aging, CD8^+^ T cells, cognitive dysfunction, murine hepatitis virus, neuroinflammation, resident memory T cells, respiratory infection

## Abstract

Advanced age increases the risk of severe disease from SARS‐CoV‐2 infection, as well as incidence of long COVID and SARS‐CoV‐2 reinfection. We hypothesized that perturbations in the aged antiviral CD8^+^ T cell response predisposes elderly individuals to severe coronavirus infection, re‐infection, and postinfectious cognitive sequelae. Using MHV‐A59 as a murine model of respiratory coronavirus, we found that aging increased CNS infection and lethality to MHV infection. This was coupled with increased CD8^+^ T cells within the aged CNS but reduced antigen specificity. Aged animals also displayed a decreased proportion of CD103^+^ resident memory cells (T_RM_), which correlated with increased severity of secondary viral challenge. Using a reciprocal adoptive transfer paradigm, data show that not only were fewer aged CD8^+^ T cells retained within the adult brain post‐infection, but also that adult CD8^+^ cells expressed lower levels of T_RM_ marker CD103 when in the aged microenvironment. Furthermore, aged animals demonstrated spatial learning impairment following MHV infection, which worsened in both aged and adult animals following secondary viral challenge. Spatial learning impairment was accompanied by increased TUNEL positivity in hippocampal neurons, suggestive of neuronal apoptosis. Additionally, primary cell coculture showed that activated CD8^+^ T cells induced TUNEL positivity in neurons, independent of antigen‐specificity. Altogether, these results show that non‐antigen specific CD8^+^ T cells are recruited to the aged brain and cause broad neuronal death without establishing a T_RM_ phenotype that confers lasting protection against a secondary infection.

## Graphical abstract

Advanced age increases CNS infection and lethality following respiratory coronavirus infection, coupled with increased infiltration of activated effector (T_eff_) CD8^+^ T cells into the CNS. Aged T_eff_ are not antigen specific, and do not effectively transition to long‐lived protective T_RM_, leaving aged mice more susceptible to secondary viral rechallenge. However, activation of T_eff_ and reactivation of T_RM_ contributes to neuronal death and postinfectious cognitive impairment in both adult and aged mice, albeit with increased severity in aged individuals.

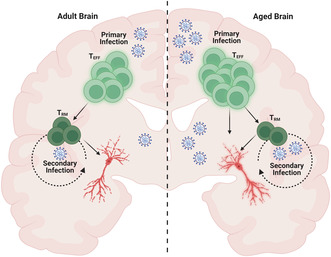

**Abbreviations**
ANOVA: Analysis of Variance
APC: Allophycocyanin
APC‐Cy7: Allophycocyanin‐Cyanine 7
AUC: Area under the curve
BBB: Blood‐Brain Barrier
BV: Brilliant Violet
CA1: Cornu Ammonis 1
CA3: Cornu Ammonis 3
CLN: Cervical Lymph Node
CNS: Central Nervous System
COVID: Coronavirus Disease
DAPI: 4′,6‐diamidino‐2‐phenylindole
DCX: Doublecortin
DG: Dentate Gyrus
DPC: Days Post‐Challenge
DPI: Days Post‐Infection
FBS: Fetal Bovine Serum
FITC: Fluorescein Isothiocyanate
GZMB: Granzyme B
HBSS: Hanks' Balanced Salt Solution
i.c.: Intracranial
IgG: Immunoglobulin G
i.n.: Intranasal
IFN‐γ: Interferon gamma
IHC: Immunohistochemistry
IL‐1β: Interleukin 1 beta
IL‐6: Interleukin 6
MdLN: Mediastinal Lymph Node
MFI: Mean Fluorescence Intensity
MHC: Major Histocompatibility Complex
MHV: Murine Hepatitis Virus
MOI: Multiplicity of Infection
MPEC: Memory Precursor Effector Cells
mRNA: Messenger RNA
PBS: Phosphate Buffered Saline
PCC: Pearson Correlation Coefficient
PE: Phycoerythrin
PE‐Cy7: Phycoerythrin‐Cyanine 7
PerCP: Peridinin‐Chlorophyll‐Protein Complex
PerCP‐Cy5.5: Peridinin‐Chlorophyll‐Protein Complex‐Cyanine 5.5
PFA: Paraformaldehyde
pfu: Plaque Forming Units
PMA: Phorbol 12‐myristate 13‐acetate
qRT‐PCR: Quantitative Reverse Transcription Polymerase Chain Reaction
SARS‐CoV‐2: Severe Acute Respiratory Syndrome Coronavirus 2
SLEC: Short Lived Effector Cells
TNF: Tumor Necrosis Factor
Teff: Effector T cells
TRM: Tissue Resident Memory T cells
TUNEL: Terminal deoxynucleotidyl transferase dUTP nick end labeling
WNV: West Nile virus

## 1 INTRODUCTION

Increasing evidence correlates exposure to viral infections with cognitive dysfunction and neurodegenerative diseases (Lotz et al., 2021). Neurotropic viruses such as Zika virus, West Nile virus (WNV), and herpes simplex virus have long been associated with postinfectious cognitive sequelae attributed to immune cell infiltration within the brain (De Chiara et al, 2019; Figueiredo et al., 2019; Fulton et al., 2020). Recent data suggest that exposure to viral respiratory pathogens, including influenza, respiratory syncytial virus, and SARS‐CoV‐2 may cause neurologic symptoms and postinfectious cognitive decline (Andrade et al., 2022; Ellul et al., 2020; Jurgens et al., 2012). While SARS‐CoV‐2 manifests primarily in the lower respiratory tract as viral pneumonia, 36% of patients develop neurologic symptoms of disease including fatigue, myalgia, headache, anxiety and depression, some of which persist long after resolution of acute viral infection, known as “long COVID” (Taquet et al., 2021). Long COVID was initially recognized in patients with severe infections, but recent reports have described these symptoms in patients with relatively mild illness (Méndez et al., 2022). Whether the symptoms of long COVID are due to direct viral invasion per se or result from antiviral responses is unclear (Klein, 2022). Viral RNA has been detected in brain tissue of human patients upon autopsy (Stein et al., 2022), but this may be nonreplicating virus or rare occurrences that are not representative of the population at large (Klein, 2022). Even in the absence of direct CNS invasion, SARS‐CoV‐2 infection is characterized by elevated inflammatory cytokines IL‐1β, IL‐6, and TNF in the plasma (Arunachalam et al., 2020) and CNS infiltration of anti‐viral immune cells, including CD8^+^ T cells (Schwabenland et al., 2021), which can contribute to postinfectious cognitive disease (Reagin & Funk, 2022). With more than 600 million people worldwide who have survived COVID acute infection, it is critical that we understand the biological basis of post‐acute sequelae, including neurologic dysfunction.

Advanced age is a risk factor for severe outcomes of many viral infections, including SARS‐CoV‐2. Individuals over the age of 65 display increased incidence of severe disease and re‐infection with SARS‐CoV‐2, as well as incidence of long COVID and neurologic complications (Michlmayr et al., 2022; Sullivan & Fischer, 2021). Age‐associated changes in the antiviral immune response includes an enhanced inflammatory profile termed “inflammaging” that correlates with increased vascular permeability and basal levels of neuroinflammation (Erickson & Banks, 2019). Collectively, these changes increase viral access to the CNS that promote neurotropic infections while also impairing antiviral immune responses. With age CD8^+^ T cell responses that are necessary for viral recovery shrink in number, diversity, and quality (Funk et al., 2021; Goplen et al., 2021); however, whether age‐associated alterations in the anti‐viral CD8^+^ T cell response contribute to postinfectious cognitive impairment following coronavirus infection remains unclear.

Recurring viral infections are controlled by a persistent population of memory CD8^+^ T cells lodged permanently within the tissue known as tissue resident memory cells (T_RM_) (Schenkel & Masopust, 2014). T_RM_ are characterized by increased expression of integrins CD103 and CD69 that promote epithelial adherence and tissue retention, respectively (Szabo et al., 2019). Their presence at portals of viral entry allow rapid elimination of new viral reservoirs and can provide life‐long protection against viral infections (Hobbs et al., 2017). It is well established that T_RM_ can persist in the brain following neurotropic viral infection, where they prevent reactivation of herpes virus from latency (Khanna et al., 2003). While critical for viral control, persistence of brain resident T_RM_ can also contribute to postinfectious cognitive sequelae, which can have debilitating long‐term consequences for the host (Garber et al., 2019; Reagin & Funk, 2022). In addition to reduced activation of acute antiviral CD8^+^ T cell responses, aging is associated with impaired T_RM_ formation due to many factors including a depleted pool of naïve T cells, impaired proliferation, and the inflammatory microenvironment (Nikolich‐Žugich, 2018).

We hypothesized that the increased susceptibility of aged individuals to both severe and recurring viral infections may be attributed to (1) failure of aged immune responses to elicit a sufficient primary CD8^+^ T cell response to control viral replication, and (2) failure of aged CD8^+^ T cells to establish as long‐lived protective T_RM_. To examine the effect of advanced age on coronavirus‐specific CD8^+^ T cell responses and T_RM_ formation in the brain, we established a mouse model of respiratory coronavirus infection using mouse hepatitis virus strain A59 (MHV‐A59). Similar to SARS‐CoV‐2, MHV‐A59 is a member of the subgroup 2a β‐coronavirus clade; however, MHV‐A59 is a natural mouse pathogen that readily infects wildtype mice (Körner et al., 2020). Using this model of respiratory coronavirus infection, we found that advanced age significantly increased clinical illness, lethality, and viral titers in the CNS during acute infection. This increase in CNS viral burden in aged animals was coupled with spatial learning impairment and heightened infiltration of activated CD8^+^ T cells into the CNS. Despite harboring numerically more activated CD8^+^ T cells, a lower proportion of CD8^+^ T cells infiltrating the aged brain were specific to MHV or expressed markers of long‐term protective CD8^+^ T_RM_. Using an adoptive transfer model, we show that aged CD8^+^ T cells were intrinsically defective in their ability to establish in the CNS. Furthermore, reduced presence of antigen‐specific CD8^+^ T cells within the brain of aged animals resulted in increased disease severity upon secondary viral challenge. Both adult and aged animals demonstrated neuronal death within the hippocampus following primary infection in vivo and in primary neuron cultures and worsened with secondary viral challenge and T_RM_ reactivation. Altogether these data suggest that CD8^+^ T cells nonspecifically infiltrate the CNS in aged animals following respiratory coronavirus infection and contribute to postinfectious cognitive decline via neuronal death but are impaired at establishing long‐lasting protective T_RM_.

## 2 RESULTS

### 2.1 Aged mice are more susceptible to respiratory coronavirus infection

**FIGURE 1:**
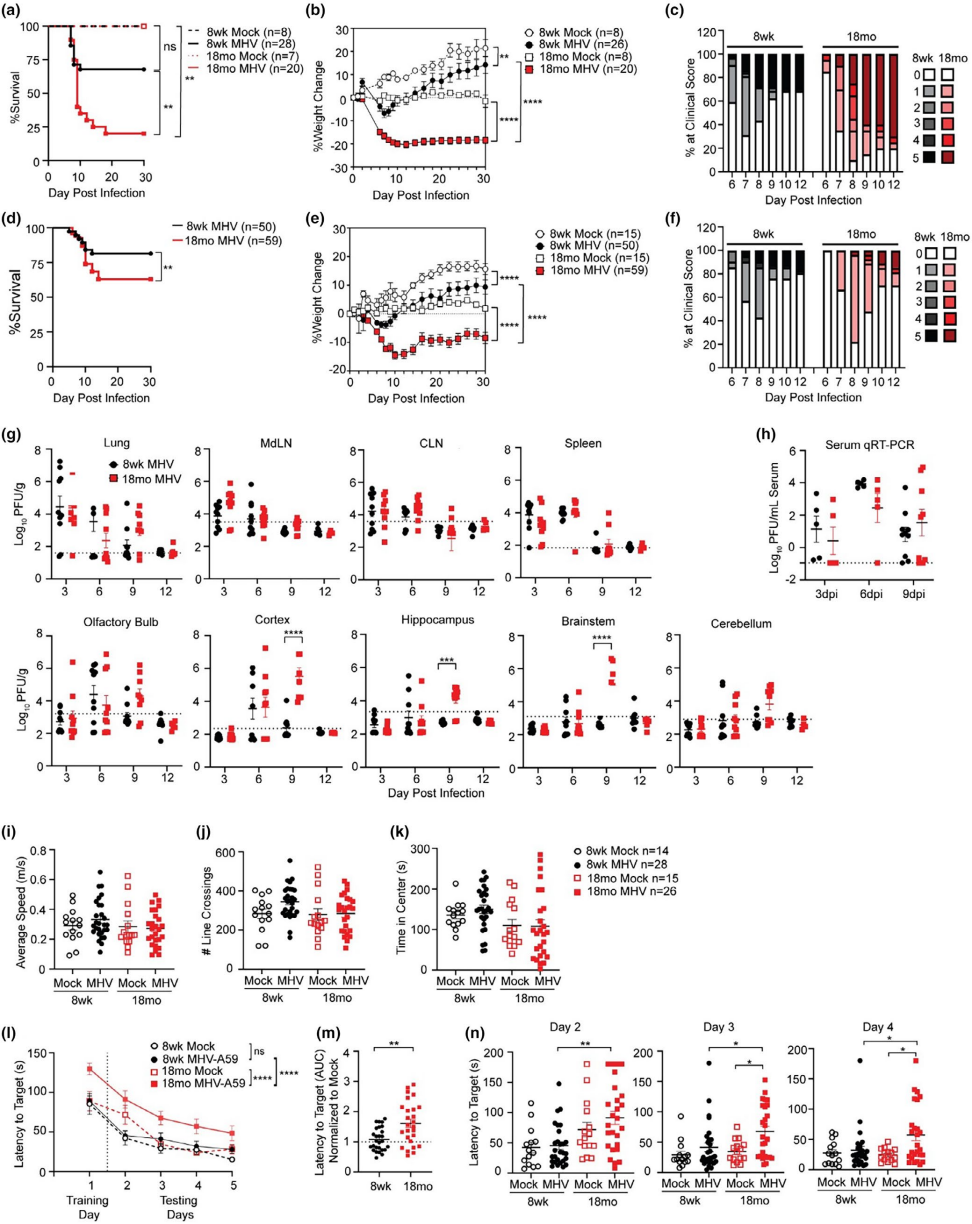
Aged mice are more susceptible to severe respiratory coronavirus infection. Eight week adult or 18 months aged C57BL/6 mice were inoculated with (a–c) 10^4^ pfu or (d–j) 10^3^ pfu MHV‐A59 or HBSS (mock infected) i.n. then monitored for (a, d) mortality, (b, e) weight change, and (c, f) clinical illness for 30 DPI. Clinical scores during acute stages of illness were scored as 0 = subclinical, 1 = hunched/ruffled fur, 2 = altered gait/slow movement, 3 = responsive to stimuli, 4 = moribund, 5 = dead. Data were pooled from 3 independent experiments with the sample size indicated. (g) Viral loads were quantified by plaque assay on the indicated DPI with 10^3^ pfu MHV‐A59 i.n. (h) Serum viral loads were determined by qRT‐PCR. (i–n) Anxiety and spatial learning assessment of 8 week adult or 18 months aged C57BL/6 mice following recovery from 10^3^ pfu MHV‐A59 or HBSS (mock) treatment. (i–k) Open field assessment was conducted at 24 DPI and measured the (i) average speed, (j) total number of lines crossed, and (k) total time spent in the center region. (l–n) Barnes maze was conducted at 25–29 DPI and latency to target hole was assessed twice each day for 5 days (l). (m) Overall latency to target was normalized to mock infected age matched controls and AUC analysis conducted. (n) Latency to target by adult vs. aged, mock vs. infected animals on testing days 2, 3, and 4. Data were pooled from 4 independent experiments with each data point representing a single animal. Survival assessed by Log‐rank Mantel‐Cox assessment, weight change and viral titer assessment conducted according to two‐way ANOVA. * *p* < 0.05; **p < 0.01; ***p < 0.001; ****p < 0.0001.

While conventionally associated with hepatic pathology and transient demyelination, intranasal inoculation of MHV‐A59 into the respiratory tract elicits productive lung infection, production of proinflammatory cytokines including TNF, IL‐6, IL‐1β, and IFN‐γ, and alveolar pneumonia (Körner et al., 2020; Lavi et al., 1984; Yang et al., 2014), which models many aspects of acute respiratory distress syndrome seen in human SARS‐CoV‐2 patients. Using MHV‐A59 we examined whether advanced age impacted recovery from respiratory coronavirus infection. Eight week (adult) or 18 months old (aged) C57BL/6 male mice were infected with 10^4^ pfu MHV‐A59 via intranasal (i. n) inoculum and their survival assessed until 30 days postinfection (DPI). While 25% of adult animals succumbed to infection, lethality was significantly increased in aged animals to 80% mortality (Figure 1a). Adult animals displayed mild weight loss and clinical illness during the acute stages of infection (6–8 DPI) that resolved by 10 DPI (Figure 1b,c). MHV‐infected aged animals also showed significantly increased weight loss compared to mock‐infected aged animals or MHV‐infected adult animals and did not recover their initial weight by 30 DPI. Additionally, aged animals showed more severe clinical signs of illness during the acute stages of infection with a greater percentage of animals exhibiting clinical scores of 4 and 5 compared to their adult counterparts (Figure 1c).

To facilitate analysis of mechanisms of recovery from infection, we utilized a lower viral inoculum (10^3^ pfu). Aged mice still succumbed to infection at a higher rate than adult mice and exhibited increased weight loss and clinical illness, but these differences between adult and aged animals were lessened when inoculated with the lower viral titer (Figure 1d–f). To determine if increased disease severity observed in aged animals correlated with elevated viral load, we examined viral titers in the respiratory tract and secondary lymphoid organs following low dose viral infection. Adult and aged animals demonstrated comparable levels of replicating virus in the lung, lung draining mediastinal lymph node (MdLN), cervical lymph node (CLN), and spleen (Figure 1g), and viral genome in the serum (Figure 1h). This suggested that enhanced clinical illness in aged animals was not attributed to uncontrolled viral replication in peripheral tissues. Adult and aged animals demonstrated similar kinetics of viral dissemination to the CNS with virus appearing first in the olfactory bulb at 3–6 DPI, spreading to the cortex and hippocampus by 6 DPI, then to the hindbrain (cerebellum and brainstem) by 6–9 DPI (Figure 1g). However, aged animals harbored significantly increased levels of replicating virus within the cortex, hippocampus, and brainstem at 9 DPI, a time at which virus had been cleared from the CNS of adult animals (Figure 1g). No virus was detected in peripheral tissues or the CNS by 12 DPI, indicating that both adult and aged animals that survived acute infection successfully cleared the virus. Notably, we tested whether residual viral genome persisted beyond the 12 DPI time point by measuring MHV‐A59 N gene expression by qRT‐PCR. Comparable levels of MHV N genome was detected in the lungs, cortices, and hippocampi of adult and aged animals at 12 DPI, indicating the presence of either nonreplicating virus or replicating virus that was below the limit of detection of the plaque assays, but no viral N mRNA was detected in adult or aged animals by 30 DPI, suggesting any persisting viral reservoirs are successfully cleared by this time (Figure S1a). To determine whether MHV‐A59 infected similar targets in the brains of adult and aged mice, we performed immunohistochemical (IHC) analysis on brain tissue sections from mice at 6 DPI, a time point when virus was present in both adult and aged cortices. MHV nucleoprotein (N protein) colocalized with NeuN^+^ neurons and Iba1^+^ myeloid cells, but not GFAP^+^ astrocytes, in the prefrontal cortex of both adult and aged mice (Figure S1b). Furthermore, MHV N^+^Iba1^+^ cells consisted of both TMEM119^+^ brain‐resident microglia as well as TMEM119^neg^ brain‐infiltrating macrophages indicating that MHV infects CNS resident microglia, CNS infiltrating macrophages, and neurons of both adult and aged mice (Figure S1c). Together these data suggest that aged versus adult animals are more susceptible to severe outcomes from respiratory MHV infection, in accordance with enhanced viral infiltration into the CNS.

### 2.2 Respiratory coronavirus infection results in spatial learning defects in aged animals

To test whether respiratory MHV‐A59 infection caused cognitive impairment, mock or MHV‐A59‐recovered 8 week and 18 months old animals were subjected to open field assessment to measure locomotor function and anxiety, followed by five sequential days of testing in the Barnes maze to assess spatial learning. Open field assessment found no differences between adult or aged animals regardless of infection status in locomotor function measured by animal speed (Figure 1i) or number of line crossings (Figure 1j), or anxiety measured by the total time spent in the center of the arena (Figure 1k). However, when assessed by Barnes maze, post‐MHV‐A59 aged animals were significantly slower to find the target hole compared to either mock infected age‐matched controls or adult animals, regardless of infection status (Figure 1l). Area under the curve (AUC) analysis of adult vs. aged animals normalized to their mock controls demonstrated a significantly increased target latency by aged animals (Figure 1m). While all animals improved over the 5‐day training period, MHV‐infected aged animals were persistently slower to find the target hole, particularly on testing days 2–4 (Figure 1n). Comparatively, adult animals showed no significant difference in spatial learning following infection (Figure 1l–n), suggesting the observed postinfectious cognitive deficits were exclusive to aged animals in this infection model.

### 2.3 Aged mice have enhanced CD8^+^ T cell responses compared to adult animals following recovery from respiratory coronavirus infection

**FIGURE 2:**
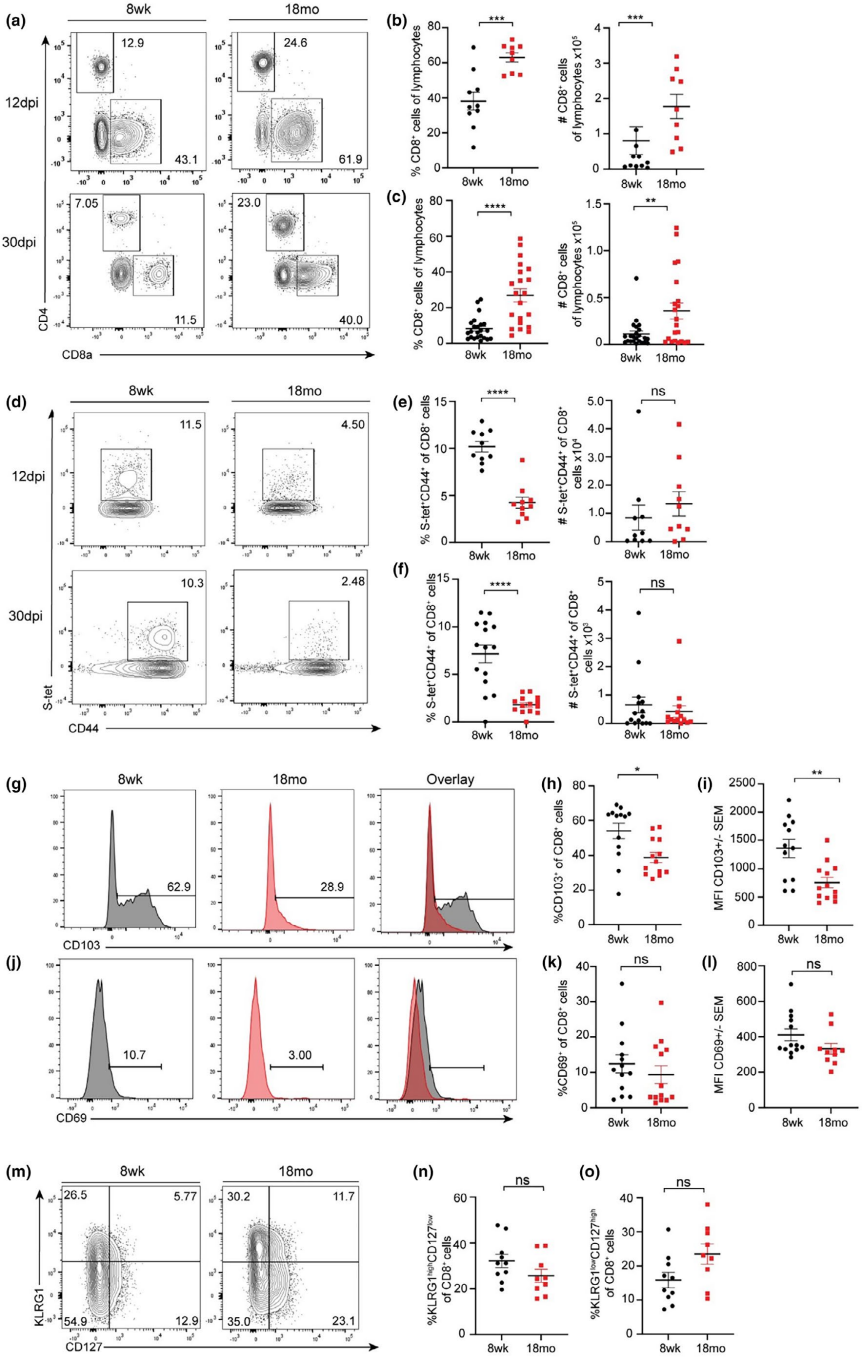
Aged animals have impaired antigen‐specific CD8^+^ T cell responses and T_RM_ formation in the CNS following respiratory coronavirus infection. Eight week adult or 18 months aged C57BL/6 mice were infected i.n. with 10^3^ pfu MHV‐A59 and CD4^+^ and CD8^+^ T cell response were assessed in the cortex at 12 and 30 DPI. (a) Representative flow cytometry plots of lymphocytes stained for CD4^+^ and CD8^+^ in the brain 12 DPI (top) and 30 DPI (bottom). (b, c) Quantification of the frequency and total number of CD8^+^ T cells at each time point. (d) Representative flow cytometry plots of CD44 and MHV S‐tetramer on CD8^+^ T cells from the cortices of adult or aged animals at 12 DPI (top) and 30 DPI (bottom). (e, f) Quantification of frequency and total number of CD44^hi^S‐tet^+^ cells. (g, j) Representative flow cytometry plots of CD103 and CD69 expression by CD8^+^ T cells from the cortices of adult (grey) or aged (red) animals at 30 DPI. Frequency of (h) CD103^+^ and (k) CD69^+^ cells. MFI of (i) CD103 and (l) CD69. (m) Representative flow cytometry plots of CD127 and KLRG1 expression by CD8^+^ T cells from the cortices of adult or aged animals at 12 DPI, with quantification of frequency of (n) KLRG1^high^CD127^low^ and (o) KLRG1^low^CD127^high^ cells. Data were pooled from 3 or 4 independent experiments with each data point representing an individual animal. Statistics according to unpaired Student T‐test. *p < 0.05; **p < 0.01; *** *p* < 0.001; ****p < 0.0001.

We wanted to determine if age‐associated alterations in the antiviral CD8^+^ T cell response were responsible for increased viral dissemination to the brain and postinfectious cognitive impairment observed in aged animals. We examined the CD8^+^ T cell response within adult and aged cortices during both acute (12 DPI) and recovery (30 DPI) stages of MHV infection by flow cytometry (Figure S2a). We found a significant increase in the infiltration of both CD4^+^ and CD8^+^ T cells into the aged brain, with the frequency and total number of CD4^+^ and CD8^+^ T cells significantly elevated in the brains of aged animals compared to adult controls at both acute and recovery time points (Figure 2a–c). This was unexpected as cellular immunity to viral infection was previously shown to be decreased in the brains of aged mice (Funk et al., 2021). However, despite harboring increased numbers of CD8^+^ T cells, a significantly lower proportion of CD8^+^ T cells within the aged brain were MHV‐specific, when assessed by MHC I tetramer against the immunodominant MHV S protein (Figure 2d). Adult animals harbored increased percentage of antigen‐specific MHV S‐tet^+^ cells compared to aged animals both acutely (11% vs. 4%) and following recovery from infection (10% vs. 2%), but because of the overall increase in total CD8^+^ cells in the aged brain, there was no difference in the number of MHV‐specific CD8^+^ T cells at either timepoint (Figure 2e,f).

The enhanced CD8^+^ T cell response observed in aged animals after MHV‐A59 infection was not restricted to the CNS, as aged animals harbored increased activated CD44^hi^CD8^+^ T cells in all sites assessed, including the lung, MdLN, CLN, and spleen at both low and high viral inoculum (Figure S2b,c). This was likely not driven by enhanced viral burden, as equivalent levels of replicating virus were detected in those peripheral sites at all time points assessed (Figure 1), and persisting viral reservoirs were cleared by 30 DPI (Figure S1a). However, enhanced blood brain barrier (BBB) disruption, which occurs with advanced age would permit nonspecific infiltration of leukocytes into the CNS (Erickson & Banks, 2019). We examined BBB permeability via immunohistochemistry of infiltrating IgG in adult and aged animals at 30 DPI. Results showed a nonsignificant increase in IgG staining in mock‐infected aged versus adult animals, and a statistically significant increase in MHV‐infected animals of both age groups (Figure S2d,e). Accordingly, aged animals showed an increased proportion, but not absolute number, of CD8^+^ T cells within the brain even in the absence of infection (Figure S2e). Together, these data suggest that BBB disruption associated with advanced age permits entry of nonspecific CD8^+^ T cells into the CNS.

### 2.4 Aged CD8^+^ T cells express lower levels of T_RM_ associated integrin CD103

CD8^+^ T cells that infiltrate peripheral tissues during acute stages of infection are referred to as effector cells (T_eff_); however, CD8^+^ T cells which persist in peripheral sites long‐term following recovery from infection are conventionally classified as T_RM_, which protect against subsequent infection (Schenkel & Masopust, 2014). As aged individuals are more susceptible to secondary coronavirus infection and aging has been shown to result in alterations in T_RM_ establishment in sites such as the lung (Goplen et al., 2020; Michlmayr et al., 2022), we wanted to determine if T_RM_ establishment was perturbed within the brain of aged animals following MHV‐A59 infection. We examined expression of integrins CD103 and CD69, which promote epithelial retention and prevent tissue egress respectively, on adult and aged CD8^+^ T cells persistent in the brain at 30 DPI. Aged CD8^+^ T cells showed a significant reduction in CD103 expression in both the frequency of CD103^+^ cells and mean fluorescence intensity (MFI) compared with adult CD8^+^ T cells, while CD69 expression was comparable irrespective of age group (Figure 2g–l). However, aged animals showed no alterations in the proportion of T_eff_ prone to survival and memory cell establishment (KLRG1^low^CD127^high^; memory precursor effector cells, MPECs) versus those prone to terminal differentiation and death (KLRG1^high^CD127^low^; short lived effector cells, SLECs) (Figure 2m–o), suggesting aged T_eff_ have the potential to develop into T_RM_ but that this process is perturbed with advanced age.

### 2.5 Aged CD8^+^ T cells are intrinsically deficient in memory establishment

**FIGURE 3:**
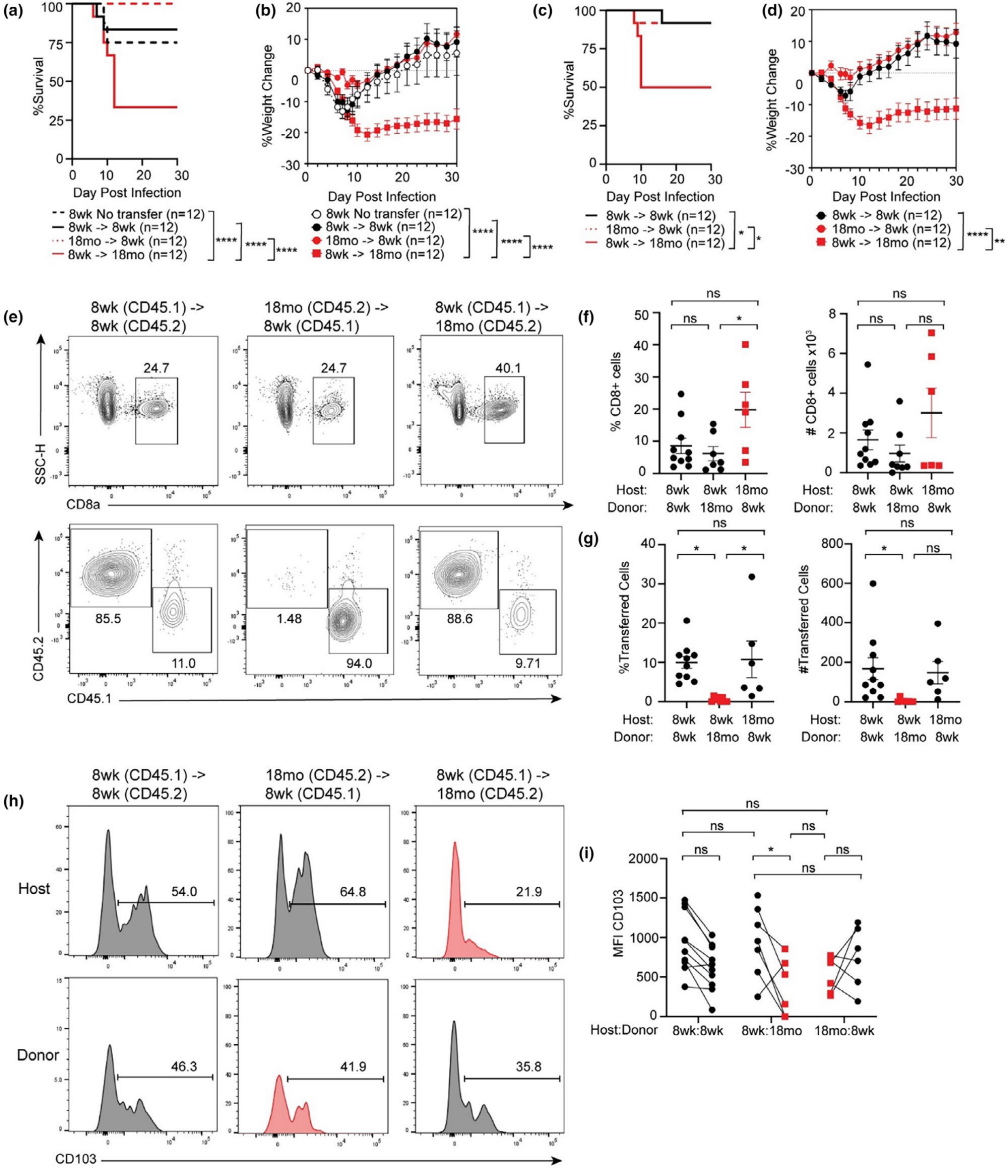
Aging limits T_RM_ establishment within the brain. 8 week. adult or 18 months aged C57BL/6 mice were inoculated with 10^3^pfu MHV‐A59 i.n., CD8^+^ T cells harvested from the spleen 7 DPI and then adoptively transferred intravenously (i.v.) via the tail vein into either 8 week. or 18 months old animals that were infected with (a,b) 10^4^ pfu or (c,d) 10^3^ pfu MHV‐A59 1 day prior. (a, c) Survival and (b, d) weight change were monitored for 30 DPI. (e) Representative flow cytometry plots of lymphocytes stained for CD8, then CD45.1 and CD45.2 to distinguish between host and donor cells isolated from the brains of 8 week. or 18 months old animals at 30 DPI following infection with 10^3^pfu MHV‐A59 and adoptive transfer of 8 week. or 18 months cells as indicated. (f) Frequency and (g) total number of total CD8^+^ cells and total transferred cells. (h) Representative flow cytometry histograms of CD103 expression by CD8^+^ T cells present in the brain of 8 week. or 18 months animals 30 DPI following infection and adoptive transfer as described. (i) Quantification of CD103 MFI by host or donor cells within the same host. Data are representative of 1 independent experiment with each data point representing an individual animal. Survival assessed by Log‐rank Mantel‐Cox assessment, weight change and flow cytometry assessment conducted according to two‐way ANOVA. *p < 0.05; ** *p* < 0.01; ***p < 0.001; ****p < 0.0001.

We next wanted to determine if the defects in CD103^+^ T_RM_ establishment in the aged brain were cell‐intrinsic or due to limitations within the aged brain microenvironment. We conducted reciprocal adoptive transfer experiments in which CD8^+^ T_eff_ were isolated from the spleens of adult or aged animals at 7 DPI 10^3^ pfu MHV‐A59 i.n. infection, then transferred i.v. into either adult or aged animals infected with 10^4^ pfu MHV‐A59 i.n. one day prior. We postulated that adoptive transfer of T_eff_ from adult donors would improve disease course of aged recipients; however, aged recipients maintained a significantly greater mortality and weight loss compared to any adult group (Figure 3a,b) at levels comparable to those observed in the absence of adoptive transfer (Figure 1a,b). This was consistent when recipient animals were infected with the lower inoculum, as aged animals that received adult CD8^+^ T_eff_ still showed increased mortality and weight loss compared to adult controls (Figure 3c,d). Although adult mice that received aged donor T_eff_ cells showed improved survival compared to those that received adult donor cells or no transfer controls, these differences were not statistically significant. This suggests that enhancing the numerical CD8^+^ T cell response in aged animals during the acute stage of infection is not sufficient to prevent lethal MHV‐A59 infection in aged mice.

We next assessed T_RM_ establishment within the brain 30 days following adoptive transfer using the lower viral inoculum recovery model. Consistent with our previous observations, aged animals that received adult T_eff_ showed increased proportion of total CD8^+^ T cells compared to adult animals that received adult or aged T_eff_ but no statistical difference in the number of CD8^+^ T cells in any age or transfer group (Figure 3e,f). We assessed the proportion of host vs. donor CD8^+^ T cells within the brains at 30 DPI using differential isoforms of the congenic marker CD45 (CD45.1 and CD45.2). In adult (CD45.2) animals which received adult (CD45.1) T_eff_, an average of 10%–15% of the total CD8^+^ T cells in the brain were donor derived, while the remaining 85%–90% were host derived (Figure 3e,g). This was similar in aged animals (CD45.2) that received adult (CD45.1) T_eff_, suggesting a comparable potential for adult T_eff_ to establish in the brains of either adult or aged hosts. Conversely, adult animals (CD45.1) that received aged (CD45.2) T_eff_ showed little to no presence of donor cells (~1%) suggesting that aged CD8^+^ T_eff_ fail to establish in the adult brain (Figure 3e,g). Furthermore, we measured expression of T_RM_ integrin CD103 on both host and donor derived CD8^+^ T cells and found comparable expression of CD103 when both host and donor cells were adult‐derived. However, when aged donor cells were transferred into an adult host, CD103 expression on a per‐cell basis (MFI) remained persistently lower in aged donor CD8^+^ T cells compared to their adult host cells (Figure 3h,i). Altogether, these data suggest that aged CD8^+^ T cells are intrinsically deficient in upregulating CD103 and establishing as T_RM_.

### 2.6 Advanced age increases susceptibility to secondary viral rechallenge

**FIGURE 4:**
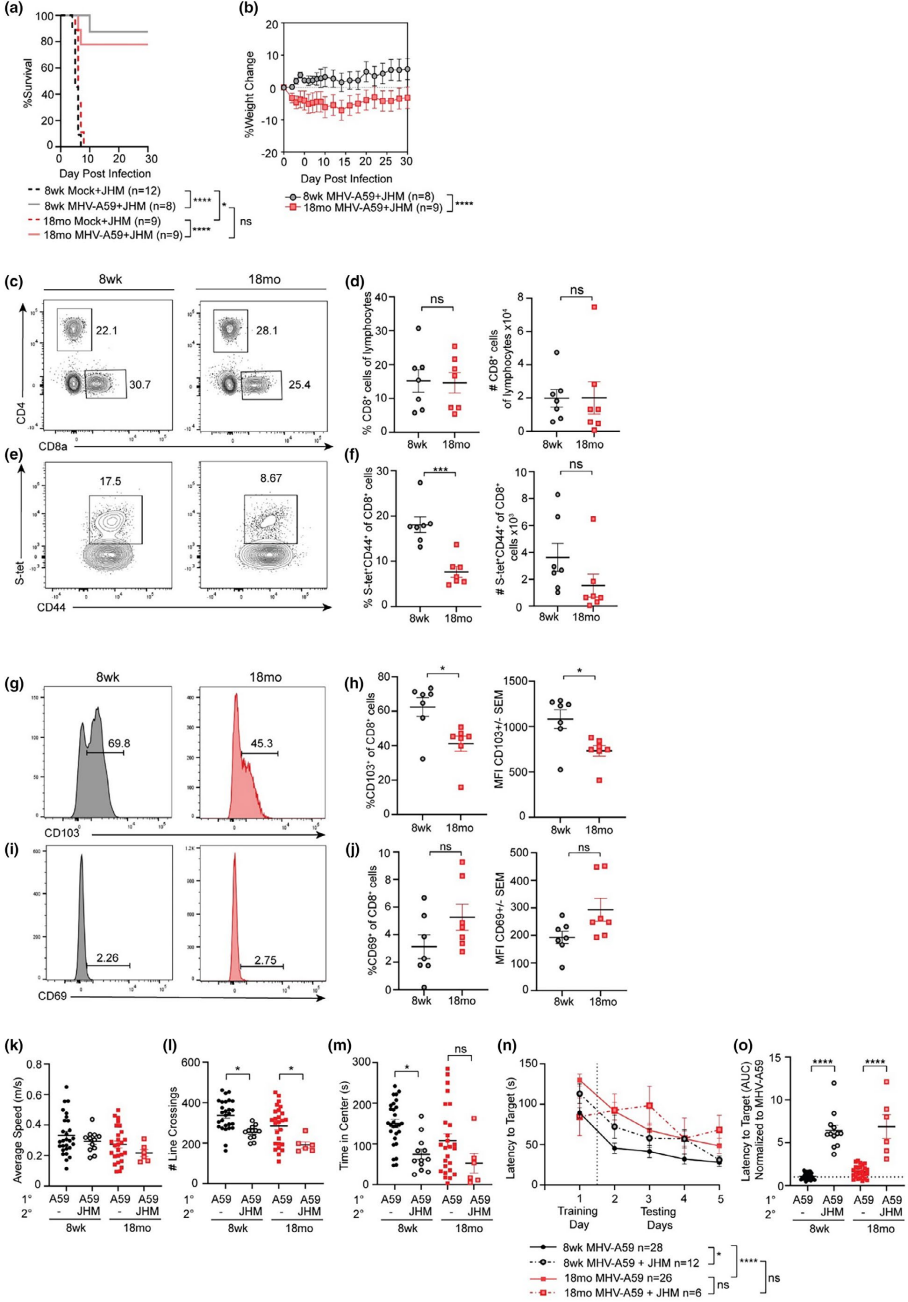
Aged animals demonstrate impaired secondary CD8^+^ T cell responses and cognitive defects upon viral rechallenge. Eight week adult or 18 months aged C57BL/6 mice were inoculated with 10^3^ pfu or HBSS (mock infected) i.n., rested for 30 days, then rechallenged with 10pfu MHV‐JHM i.c. Animals were then monitored for (a) mortality and (b) weight change for an additional 30 DPC. (c) Representative flow cytometry plots of lymphocytes stained for CD4 and CD8 isolated from the brain at 30 DPC, with (d) frequency and total number of CD8^+^ T cells quantified. (e) Representative flow cytometry plots of CD44 and MHV S‐tetramer on CD8^+^ T cells from the cortices of adult or aged animals at 30 DPC, with (f) frequency and total number of CD44^hi^S‐tet^+^ cells quantified. Representative flow cytometry histograms of (g) CD103 and (i) CD69 expression by CD8^+^ T cells from the cortices of adult (grey) or aged (red) animals at 30 DPC, with quantification of frequency and MFI of (h) CD103^+^ and (j) CD69^+^ cells. (k‐m) Open field assessment was conducted at 24 DPC/DPI to quantify (k) average speed, (l) total number of lines crossed, and (m) total time spent in the center region. (n) Barnes maze was conducted at 25–29 DPC/DPI and latency to target hole was assessed twice each day for 5 days. (o) AUC analysis of overall latency to target of rechallenged animals normalized to primary MHV‐A59 infected age matched controls. Survival assessed by Log‐rank Mantel‐Cox assessment, weight change and behavior assessment conducted according to two‐way ANOVA and flow cytometry data assessed by unpaired Student T‐test. *p < 0.05; **p < 0.01; ***p < 0.001; ****p < 0.0001.

Given that presence of antigen‐specific T_RM_ in the brain correlates with protection against secondary viral infection (Khanna et al., 2003), we hypothesized that decreased presence of antigen‐specific CD103^+^ T_RM_ within the brains of aged mice following MHV‐A59 infection would increase susceptibility to a secondary neurotropic viral infection. To assess this, we utilized a viral rechallenge model with heterologous MHV‐JHM strain. While closely related to MHV‐A59, MHV‐JHM has increased neurovirulence and is commonly used to model CNS demyelination (Wu et al., 2001). Importantly, MHV‐A59 and MHV‐JHM share a conserved immunodominant CD8^+^ T cell response, therefore memory CD8^+^ T cells generated by MHV‐A59 primary infection respond to MHV‐JHM secondary viral challenge (Bergmann et al., 1996). Adult and aged animals were inoculated i.n. with 10^3^ pfu MHV‐A59 or mock diluent, rested for 30 days, then rechallenged with 10 pfu MHV‐JHM by direct i.c. inoculation. This allowed specific assessment of the contribution of brain resident CD8^+^ memory cells, without eliciting a strong secondary viral infection within the respiratory tract. With primary inoculation, MHV‐JHM caused 100% lethality accompanied by severe neurologic manifestations of disease (hind limb splaying, shaking, scratching, righting reflex), irrespective of age (Figure 4a). However, lethality and neurologic sequalae was rescued to survival rates of approximately 80% with prior MHV‐A59 i.n. immunization, regardless of age (Figure 4a). Despite comparable levels of survival, aged animals showed significantly more weight loss over the course of MHV‐JHM rechallenge compared to their adult counterparts, suggesting that aged animals experienced more severe illness (Figure 4b). At 30 days postchallenge (DPC), we observed no significant difference in the numbers or percentage of CD8^+^ T cells in the brains of adult and aged animals, in contrast to results found with the primary MHV‐A59 infection (Figure 4c,d). However, like primary infection, the proportion of MHV‐specific S‐tet^+^CD44^hi^CD8^+^ cells was significantly reduced in aged animals compared to adult controls following MHV‐JHM rechallenge, though the total numbers of cells was not quite statistically significant (Figure 4e,f). Furthermore, aged CD8^+^ T cells again displayed decreased expression of CD103 compared to adult controls, both in the proportion of CD103^+^ cells and in MFI, though CD69 expression remained unchanged (Figure 4g–j). These results suggest that aged animals mount a limited response to viral rechallenge and further support our hypothesis that the aged brain restricts establishment of brain‐resident T_RM_.

### 2.7 Secondary viral challenge exacerbates spatial learning impairment

We next assessed whether secondary neurotropic viral challenge exacerbated spatial learning impairment compared to primary infection. We conducted open field and Barnes maze tests of adult and aged animals following primary MHV‐A59 i.n. infection and MHV‐JHM i.c. challenge at 24–29 DPI or DPC, respectively. Despite having no differences in average speed, animals challenged with MHV‐JHM displayed a significant reduction in line crossings and a reduction in time spent in the center of the arena compared to primary infected controls, irrespective of age (Figure 4k–m). This suggests an increase in anxiety behavior following secondary MHV‐JHM challenge that was not observed following primary MHV‐A59 infection (Figure 1i–k). MHV‐JHM challenged animals also demonstrated significant spatial learning impairment in the Barnes maze compared to primary infected controls of both age groups (Figure 4n,o). Adult rechallenged animals demonstrated significantly increased latency to target over the 5‐day testing period compared to adult primary infected controls, and although aged animals showed increased latency compared to adult animals following primary infection alone, secondary viral challenge did not further increase latency in aged animals according to ANOVA analysis of the full curve (Figure 4n). However, AUC analysis normalized to primary infected controls demonstrated a significant increase in target latency in rechallenged animals compared to primary infected controls in both age groups (Figure 4o). These data indicate that while primary MHV‐A59 infection caused spatial learning impairment in aged animals only, secondary infection with MHV‐JHM caused spatial learning impairment in both adult and aged animals.

### 2.8 MHV infection results in hippocampal neuronal death

**FIGURE 5:**
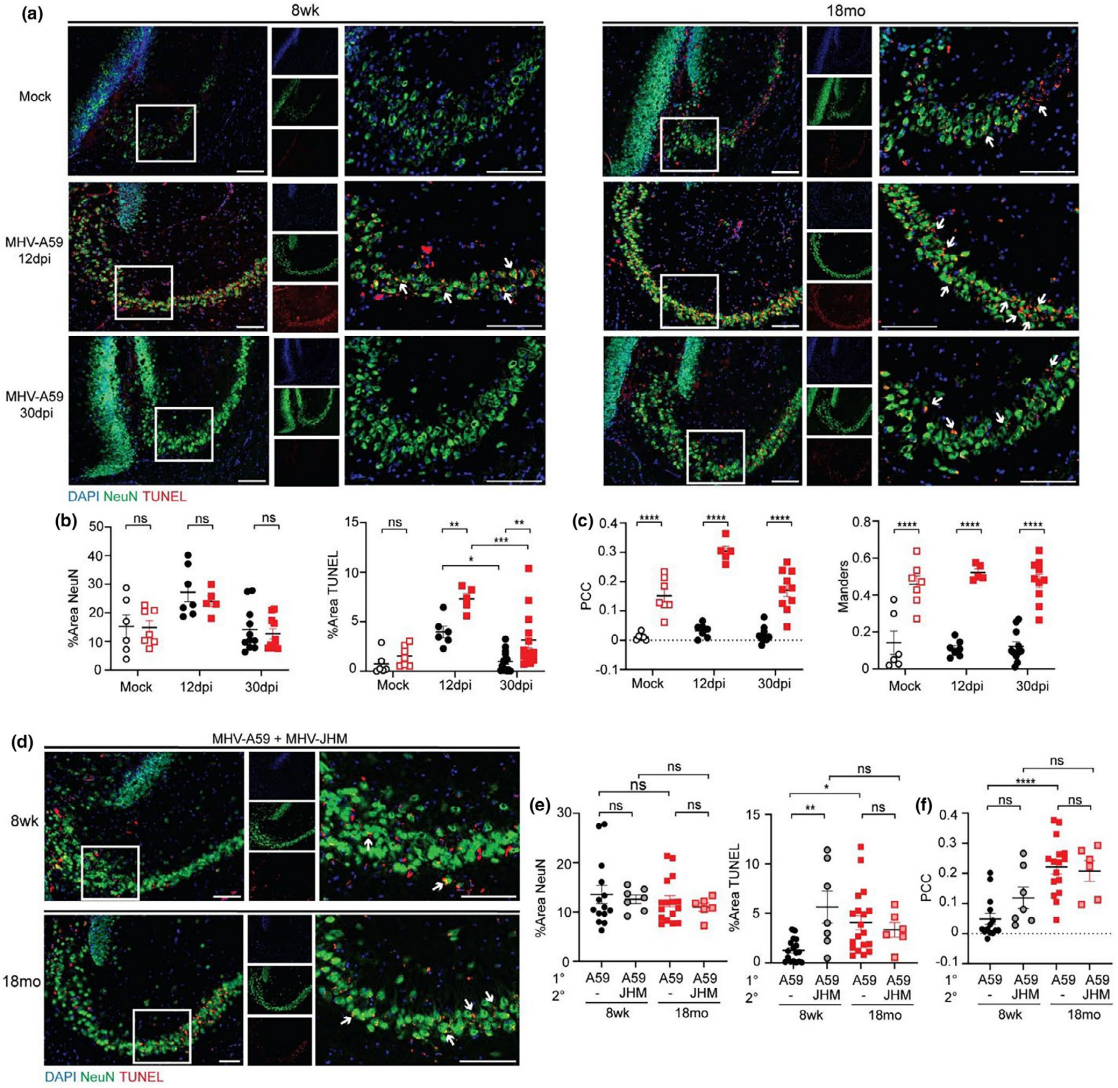
MHV‐A59 respiratory infection results in neuronal death in aged hippocampus, which worsens with secondary viral challenge. (a) Representative IHC of DAPI, NeuN, and TUNEL in the CA3 of 8 week or 18 months old animals at 12 and 30 DPI MHV‐A59 i.n. compared to mock infected controls. Images taken at 20X magnification, inset taken at 40X magnification. Scale bar = 100 μm. (b) Quantification of total percent area of NeuN and TUNEL in the CA3. (c) Colocalization between NeuN and TUNEL measured by PCC and Mander's overlap coefficient. (d) Representative IHC of DAPI, NeuN, and TUNEL in the CA3 of 8 week or 18 months old animals following primary infection (10^3^ pfu MHV‐A59 i.n., 30 DPI) and rechallenge (10 pfu MHV‐JHM i.c., as described in Figure 4, 30 DPC). Images taken at 20X magnification, inset taken at 40X magnification. Scale bar = 100 μm. (e) Quantification of total percent area of NeuN and TUNEL in the CA3 and (f) colocalization between NeuN and TUNEL compared to primary infection (MHV‐A59 i.n.) controls. Data were pooled from 2 independent experiments with each data point representing a single animal. Statistics according to unpaired one‐way ANOVA. *p < 0.05; **p < 0.01; ***p < 0.001; ****p < 0.0001.

We hypothesized that the spatial learning impairment identified by Barnes maze may have been caused by neuronal apoptosis, synapse elimination, or demyelination, all of which CD8^+^ T cells have been found to promote (Di Filippo et al., 2016; Pewe & Perlman, 2002; Shrestha & Diamond, 2007; Vasek et al., 2016; Wu et al., 2021). To first test if the spatial learning impairment observed in aged animals following primary infection correlated with neuronal apoptosis, we measured TUNEL^+^ neurons within regions of the hippocampus that are responsible for spatial learning and memory (the dentate gyrus (DG), CA1, and CA3) in adult and aged animals acutely and post recovery from primary MHV‐A59 infection. We found TUNEL^+^NeuN^+^ cells in the CA3 (Figure 5a) and DG (Figure S3a,b) in both adult and aged animals but minimal TUNEL staining in the CA1 (Figure S3e,f). More TUNEL^+^ cells were observed at the acute timepoint (12 DPI) versus the recovery timepoint (30 DPI) in both age groups, suggesting neuronal death occurs with acute infection and likely resolves with recovery. We saw more TUNEL^+^ cells in aged versus adult DG (Figure S3c) and CA3 (Figure 5b) at 12 DPI and to a lesser extent at 30 DPI. Calculating colocalization using PCC and Mander's overlap coefficient, there was significantly greater colocalization between TUNEL and NeuN in the CA3 of aged versus adult animals at all timepoints (Figure 5c) and in the DG at most timepoints (Figure S3d). Despite minimal detection of TUNEL^+^ cells in the CA1, we did observe a significant increase in TUNEL^+^ cells in the aged vs. adult CA1 at 12 DPI and increased colocalization between NeuN and TUNEL at 12 and 30dpi (Figure S3g,h); however, this was not as pronounced as in the DG or CA3. The increased neuronal death observed in aged animals was accompanied by a decrease in neurogenesis, as aged animals showed reduced doublecortin (DCX) staining in the DG compared to adult animals, regardless of infection status (Figure S3i). This suggests that MHV‐A59 infection resulted in hippocampal neuron apoptosis in both adult and aged animals acutely; however, this was more severe in aged animals and neurons were not replenished via neurogenesis.

Hippocampal neuron apoptosis was worsened with secondary viral challenge, particularly in adult animals. While adult and aged animals showed no difference in the proportion of NeuN^+^ cells within the hippocampal CA3 region following MHV‐JHM rechallenge compared to primary MHV‐A59 infection, we observed an increase in the proportion of TUNEL^+^ cells within the hippocampal CA3 of adult rechallenged mice compared to primary infected adult animals (Figure 5d,e). Aged animals exhibited high levels of NeuN^+^TUNEL^+^ co‐localization within the CA3 region following viral rechallenge, but these levels were not significantly elevated compared to primary MHV‐A59 infection (Figure 5f). This may represent sustained neuronal apoptosis from primary infection that was never repaired or the upper threshold of neuronal apoptosis that occurs during primary MHV‐A59 infection that is not superseded by secondary MHV‐JHM infection in aged animals.

While CD8^+^ T cells can elicit neuronal apoptosis, enhanced microglial activation can also have direct neurotoxic effects. Activated microglia phagocytose local synapses, resulting in cognitive impairment in both infectious and sterile models of neuroinflammation (Di Filippo et al., 2016; Vasek et al., 2016). We assessed microglial activation in adult and aged animals at baseline and following MHV‐A59 infection by flow cytometry (gating strategy exemplified in Figure S2a). While microglia activation was enhanced in aged animals at baseline and following both primary MHV‐A59 (Figure S4a–k) and secondary MHV‐JHM infections (Figure S4l–p), we observed no signs of microglia mediated synaptic elimination in our system (Figure S5a–c). Additionally, because both MHV‐A59 (Lavi et al., 1984) and MHV‐JHM (Wu et al., 2001) can cause CNS demyelination when inoculated i.c., we measured corpus callosum width using Luxol fast blue staining (Figure S5d,e, h,i) and myelin density by myelin basic protein immunohistochemistry (Figure S5f,g). We found no significant differences in any measure irrespective of age or infection status. Together, this suggests that neither microglia‐mediated synaptic loss nor demyelination significantly contributed to the spatial learning deficits reported here, and further support the hypothesis that neuronal apoptosis within the hippocampus was a primary correlate of postinfectious cognitive dysfunction in this system.

### 2.9 Activated CD8^+^ T cells mediate neuronal death in vitro

**FIGURE 6:**
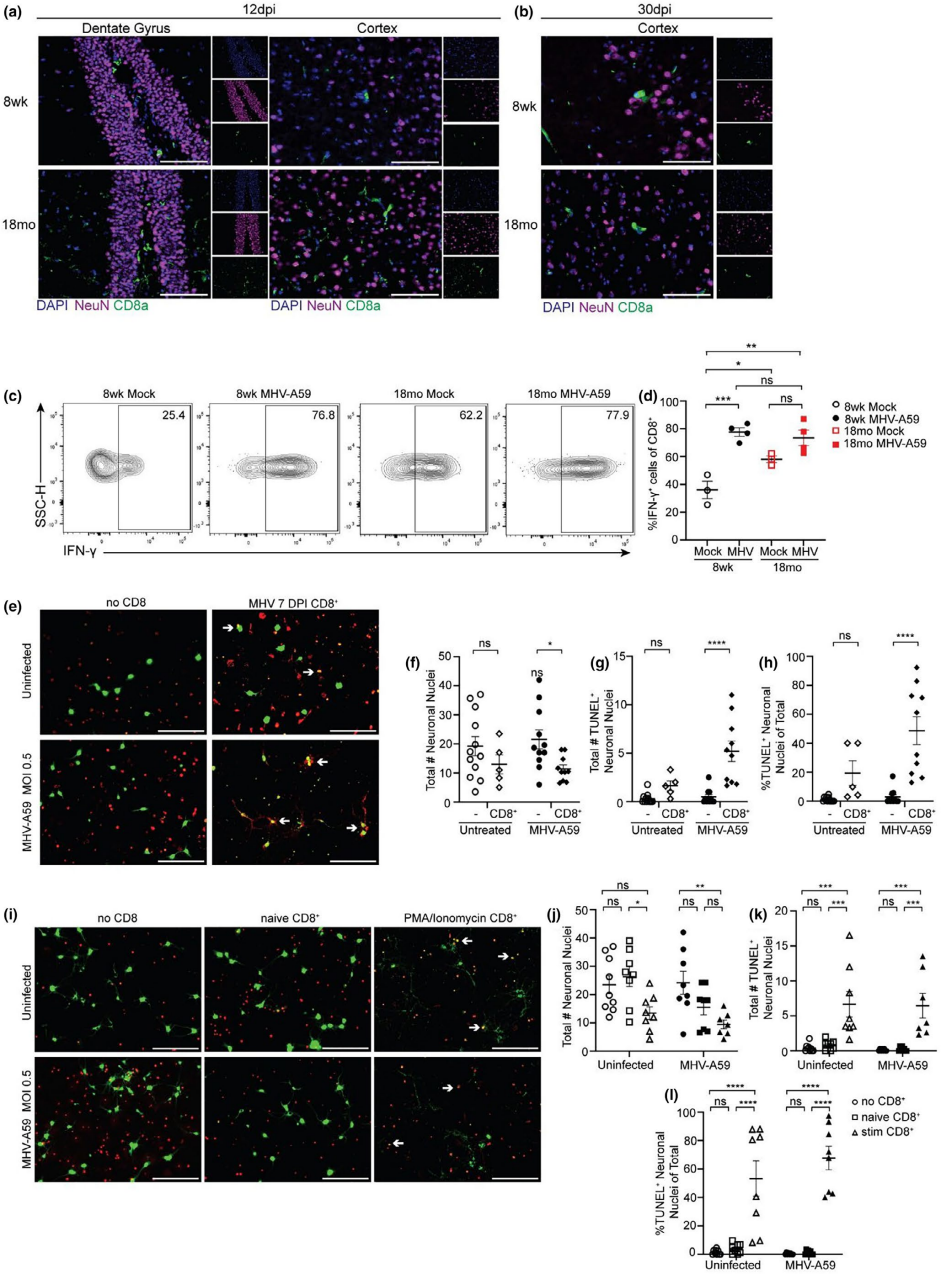
CD8^+^ T cells mediate neuronal apoptosis following MHV‐A59 infection. (a) Representative IHC of DAPI, NeuN, and CD8a in the DG and cortex of 8 week or 18 months old animals at (a) 12 DPI and (b) 30 DPI. (c) Representative flow cytometry plots of CD45^+^CD8^+^ cells stained for IFN‐γ isolated from the brains of 8 week or 18 months old, mock or MHV‐A59 infected animals at 30 DPI following 4 h. PMA/ionomycin stimulation. (d) Quantification of percent CD8^+^CD44^+^ cells positive for IFN‐γ. (e) Representative immunocytochemical (ICC) staining for NeuN and TUNEL of primary cortical neurons following no treatment or infection with MHV‐A59 MOI 0.5, with or without coculture with CD8^+^ T cells purified from the spleen of an 8 week old animal at 7 DPI with 10^3^ pfu MHV‐A59. Quantification of ICC images: (f) total number NeuN^+^ neuronal nuclei, (g) total number TUNEL^+^NeuN^+^ neuronal nuclei, (h) proportion of TUNEL^+^NeuN^+^ neuronal nuclei of total NeuN^+^ neuronal nuclei present. (i) Representative ICC of NeuN and TUNEL staining of primary embryonic cortical neurons following no treatment or infection with MHV‐A59 MOI 0.5, with or without naïve or PMA/ionomycin stimulated CD8^+^ T cells purified from the spleen of an 8 week old uninfected animal. Quantification of ICC images: (j) total number NeuN^+^ neuronal nuclei, (k) total number TUNEL^+^NeuN^+^ neuronal nuclei, and (l) proportion of TUNEL^+^NeuN^+^ neuronal nuclei of total NeuN^+^ neuronal nuclei present. Data are representative of three independent experiments with each data point representing an individual sample. All images were taken at 40X magnification and scale bar = 100 μm. Three images were captured per sample and averaged. NeuN^+^ and TUNEL^+^NeuN^+^ neuronal nuclei quantified in ImageJ software using the Cell Counter plugin. Statistics according to unpaired one‐way ANOVA. *p < 0.05; **p < 0.01; ***p < 0.001; ****p < 0.0001.

To determine if CD8^+^ T cells directly contributed to neuronal death following viral infection/rechallenge, we examined localization of brain‐infiltrating CD8^+^ T cells following primary MHV‐A59 infection. CD8^+^ T cells were easily identified at 12 DPI in the cortex and DG in both adult and aged brains via IHC (Figure 6a). By 30 DPI CD8^+^ T cells were absent from the hippocampus entirely and were only found in the cortex of adult and aged animals (Figure 6b). To further examine the cytotoxic potential of CNS resident CD8^+^ T cells, we examined production of inflammatory cytokines (IFN‐γ and TNF) and cytotoxic granules (GZMB and perforin) by CD8^+^CD44^+^ T cells isolated from the adult and aged brains of MHV‐infected or mock infected controls at 30 DPI following nonspecific stimulation with PMA/ionomycin. Given our finding that a large percentage of infiltrating CD8^+^ T cells in the aged brain were not specific to the MHV immunodominant peptide, we assessed the cytotoxic effect of CD8^+^ T cells activated in both antigen‐specific and nonspecific manners. As expected CD8^+^CD44^+^ T cells isolated from the brains of MHV‐infected adult mice had a higher proportion of IFN‐γ (Figure 6c,d) and TNF (Figure S6a,d) producing cells compared to their mock infected counterparts following stimulation with PMA/Ionomycin. Notably, the proportion of IFN‐γ producing cells was elevated in aged animals even at basal levels and increased moderately with infection (Figure 6c,d). There was no significant alterations in production of TNF, granzyme B (GZMB), or perforin between adult or aged animals irrespective of infection status (Figure S6b,c,e,f). Together, this suggested that highly activated, inflammatory, CD8^+^ T cells are elevated in response to both aging and infection.

To assess the ability of CD8^+^ T cells to induce neuronal death, we used an in vitro coculture system in which CD8^+^ T cells were isolated from the spleens of adult animals at 7 DPI MHV‐A59 infection, then cocultured with primary cortical neurons that had been inoculated with MHV at MOI 0.5 or media alone 24 h prior. Neurons and T cells were cultured together for 24 h, at which point neurons were assessed for TUNEL staining, indicative of apoptosis. We quantified the total number of NeuN^+^ neuronal nuclei as well as the total number of TUNEL^+^NeuN^+^ neuronal nuclei present in each of our culture conditions. Infection with MHV alone did not cause neuronal death as assessed by TUNEL staining, as there was no change in the total number of NeuN^+^ neurons, and we detected minimal TUNEL staining in either uninfected or MHV‐infected neurons (Figure 6e–g). However, co‐culturing infected neurons with 7 DPI CD8^+^ T cells reduced the total number of NeuN^+^ cells present in culture (Figure 6f), while the total number of TUNEL^+^NeuN^+^ cells (Figure 6g), and proportion of TUNEL^+^NeuN^+^ cells (Figure 6h) significantly increased, suggesting neuronal apoptosis. To test whether this CD8^+^ T cell mediated neuronal killing was antigen specific, we isolated CD8^+^ T cells from an uninfected animal, then stimulated them ex vivo with PMA/ionomycin to induce high levels of nonspecific activation. Stimulated CD8^+^ T cells caused significant neuronal death while uninfected/unstimulated CD8^+^ T cells did not, independent of whether cocultured neurons were infected (Figure 6i–l). This suggests that activated CD8^+^ T cells caused neuronal apoptosis, independent of CD8^+^ T cell antigen specificity or neuronal cell infection. Together, these data suggest that enhanced recruitment of activated CD8^+^ T cells into the aged CNS following neurotropic coronavirus infection can elicit neuronal apoptosis, which may contribute to the spatial learning deficits observed in aged individuals.

## 3 DISCUSSION

Approximately one in three patients who have recovered from SARS‐CoV‐2 infection experience persistent symptoms of long COVID, including cognitive complications (Taquet et al., 2021). Long COVID disproportionately affects individuals of advanced age, putting this population at a higher risk for not only severe acute infection but also persistent postinfectious symptoms (Taquet et al., 2021). To study the impact of age on respiratory coronavirus infection, we established a murine model of infection using MHV‐A59. Our results showed enhanced mortality concurrent with viral dissemination to the CNS in aged versus adult mice. Consistent with patient reports of exacerbated cognitive decline in aged individuals (Sullivan & Fischer, 2021), aged mice showed increased spatial learning impairment following MHV‐A59 infection. Aged animals in our study showed delayed spatial learning even in the absence of infection, suggesting that aging‐intrinsic factors contribute to spatial learning deficits that are exacerbated with infection, in agreement with previous publications (Yang et al., 2019). This study used exclusively male mice as early patient reports suggested aged men were the most susceptible to severe viral infection (Bwire, 2020). However, more recent reports indicate that women are at greater risk of developing long COVID (Bai et al., 2022), possibly due to their enhanced immune response, highlighting a limitation of this study and a need for specific research on sex differences in this system. While primary MHV‐A59 infection elicited spatial learning impairment only in aged animals, viral challenge with the neurovirulent MHV‐JHM strain resulted in severe manifestations of cognitive disease in all animals irrespective of age. These results are consistent with human patient data indicating that reinfection with SARS‐CoV‐2 causes an added risk of death, hospitalization, and post‐acute sequelae including pulmonary and neurologic systems, independent of vaccination status (Bowe et al., 2022). However, it is of note that murine infection with MHV results in direct viral infection of CNS cell populations including neurons and microglia, which differs from SARS‐CoV‐2 patient reports. While SARS‐CoV‐2 genome has been detected within the CNS of infected patients (Stein et al., 2022), actively replicating virus has not been recovered from this site. Therefore, we cannot eliminate the possibility that direct viral infection and lysis of CNS cell populations may contribute to spatial learning impairment observed in our model system. Furthermore, viral replication in the CNS of our model system may enhance the immune cell infiltration within the CNS compared to individuals infected with SARS‐CoV‐2. However in concordance with our results, SARS‐CoV‐2 infection elicits high systemic levels of inflammatory cytokines IL‐1β, IL‐6, and TNF in the plasma (Arunachalam et al., 2020). These high levels of inflammatory cytokines may drive immune cell recruitment to the CNS, which is supported by the observations of CD8^+^ T cells in the brains of COVID patients (Schwabenland et al., 2021). Therefore, it remains important to understand how continual viral exposure may compromise long‐term cognitive health over the course of a lifetime.

Our study provides further evidence for the role of antiviral CD8^+^ T cells as drivers of postinfectious cognitive decline. CNS infiltrating CD8^+^ T cells are necessary to protect against lethal neurotropic infection (Shrestha & Diamond, 2004), but also directly contribute to postinfectious cognitive decline via production of IFN‐γ, which can enhance microglial mediated synaptic elimination (Garber et al., 2019). Results of this study showed no evidence of synapse elimination by microglia or demyelination within cortical regions, which has been associated with neurotropic coronavirus infection previously. Rather, we found that infiltration of activated CD8^+^ T cells into the CNS was accompanied by TUNEL staining in neurons within the hippocampal trisynaptic circuit that is critical for visuospatial memory (Basu & Siegelbaum, 2015). This elevated TUNEL staining, suggesting neuronal cell death, occurred in tandem with reduced neuronal regeneration in aged but not adult animals. While we observed neuronal death in adult animals acutely, sustained neuronal regeneration during the recovery phase may restore hippocampal trisynaptic circuitry and ameliorate perturbations in visuospatial memory.

Compared to previous studies that demonstrated impaired antiviral T cell responses within the brain following neurotropic infection (Funk et al., 2021), aged animals in this study demonstrated enhanced infiltration of both CD4^+^ and CD8^+^ T cells into the CNS acutely and following recovery from MHV‐A59 infection. Despite the greater numbers of infiltrating CD8^+^ T cells, the majority of CNS infiltrating CD8^+^ T cells in the aged brain were not antigen‐specific to the immunodominant MHV S‐protein, and a reduced proportion of aged CD8^+^ T cells expressed integrin CD103, which is associated with long‐term epithelial retention as T_RM_. This reduced antigen‐specific response, and reduced CD103 expression was maintained following secondary viral challenge, suggesting a conserved mechanism regulates CD8^+^ T cell responses in aged animals following both primary and secondary MHV infection. While skin T_RM_ have shown to maintain their function even in elderly individuals (Koguchi‐Yoshioka et al., 2021), aged lung T_RM_ are ineffective in conferring protection against secondary viral challenge, and their reactivation actually drives chronic pneumonia and lung pathology (Goplen et al., 2020). Therefore, establishment and functionality of T_RM_ may differ with host age and tissue site. Considering that aging is associated with an increase in low grade systemic inflammation even under homeostatic conditions (Goplen et al., 2021), and heightened inflammatory signaling drives memory precursors to a terminally differentiated cell fate (Joshi et al., 2007), it is likely that the enhanced inflammatory microenvironment within the aged brain, coupled with enhanced BBB leakiness, could work two‐fold to drive nonspecific recruitment of CD8^+^ T cells into the CNS following infection and limit establishment of T_RM_ in this site. However, change of microenvironment was not sufficient to restore memory potential in aged CD8^+^ cells, as aged cells failed to establish as CD103^+^ T_RM_ in the brains of young hosts. This suggests that both the tissue microenvironment and inherent fate potential of individual cells play a collective role in T_RM_ establishment in aged individuals.

Despite decreased retention of MHV‐specific CD8^+^ T_RM_ within the brain of aged animals, primary infection with MHV‐A59 improved survival of both adult and aged animals when challenged with secondary infection by the neurovirulent MHV‐JHM. However, the lapse in memory formation in aged animals resulted in a more severe secondary infection course. While our analyses focused on the CD8^+^ T cell responses, other memory cell populations including memory CD4^+^ T cells, or antibody production by memory B cells may also confer protection. Interestingly, despite recovering from the MHV‐JHM challenge infection, both adult and aged animals demonstrated increased spatial learning impairment following viral rechallenge compared to primary MHV‐A59 infection alone. This was particularly interesting in the context of adult animals, which showed significant spatial learning impairment following rechallenge that was not observed following primary infection. As secondary viral infection results in T_RM_ reactivation to an effector cell‐like inflammatory state (Schenkel & Masopust, 2014), it is possible that reactivation of CNS T_RM_ following secondary viral challenge exacerbated hippocampal neuronal death and postinfectious cognitive decline in adult animals. Comparatively, aged animals showed high CD8^+^ T cell retention but low antigen‐specificity, coupled with significant spatial learning impairment, indicating that nonspecifically activated CD8^+^ T cells in aged animals also elicit hippocampal neuronal apoptosis in this system. Our data using in vitro neuron:CD8^+^ T cell cocultures further demonstrated that activated CD8^+^ T cells can induce neuronal death even in the absence of a viral cognate antigen. While CD8^+^ T cells isolated from an MHV‐A59 infected mouse at 7 DPI induced apoptosis of MHV‐A59 infected neurons, CD8^+^ T cells isolated from an uninfected mouse, when stimulated with PMA/ionomycin, also caused neuronal death to both MHV‐A59 infected and uninfected primary neurons. This suggests that presence of any activated CD8^+^ T cells within the CNS (including reactivated T_RM_ or infiltrating T_eff_) can induce neuronal apoptosis of both infected target cells and uninfected bystander cells independent of antigen specificity.

Our results point to CD8^+^ T cells as mediators of neuronal death following MHV infection. We observed heightened levels of CD8^+^ T cell activation and cytokine production, including IFN‐γ and TNF, following infection in young animals compared to mock infected controls. However, CD8^+^ T cells within the brain of aged animals showed elevated levels of IFN‐γ production irrespective of infection status. Aged CD8^+^ T cells have a lower threshold of T cell receptor engagement requisite for successful activation, suggesting that aged CD8^+^ T cells maintain a basal level of heightened responsiveness to inflammatory signals (Goronzy et al., 2012). This, coupled with the increased basal levels of inflammation that occur with age, suggests that aged CD8^+^ T cells are poised in a constant inflammatory state that is harmful to neurons upon their entry into the CNS (Goplen et al., 2021). Adult and aged CD8^+^ T cells also displayed increased expression of GZMB following MHV‐A59 infection, albeit not to statistically significant levels; therefore, it is likely that IFN‐γ may act in concert with other inflammatory mediators to contribute to neuronal death in our infection model. While previous studies have shown that high levels of IFN‐γ can negatively impact neuronal health, including regulating neurotoxicity, and neurogenesis (Li et al., 2010; Mizuno et al., 2008), future experiments will be required to determine the direct mechanisms of CD8^+^ T cell induced neuronal apoptosis in our system and how those may be exacerbated with advanced age.

The data presented here establish a role for CD8^+^ T cells in postinfectious cognitive decline following respiratory coronavirus infection. These data demonstrate alterations in the antiviral CD8^+^ T cell response in aged animals, which may underlie the severity of coronavirus infection observed in elderly patients. In conclusion, we show that alterations in the aged immune response to MHV‐A59 coronavirus infection may contribute to cognitive disease and may inform improved therapeutic interventions to SARS‐CoV‐2 and other coronavirus infections.

## 4 METHODS

### 4.1 Virus preparation

MHV‐A59 (VR‐764) stock and producer cell line NCTC‐1469 (CCL‐9.1) were obtained from the American Type Culture Collection, ATCC. MHV‐JHM (NR‐53718) stock and producer cell line 17Cl‐1 (NR‐53719) were obtained from BEI Resources. NCTC‐1469 were maintained in Dulbecco's modified Eagle's Medium (DMEM; Life Technologies, 11,965,126) supplemented with 10% heat inactivated horse serum (VWR, 103219–558) and 2 mM L‐glut (Life Technologies, 35,050–061). 17Cl‐1 cells were maintained in DMEM supplemented with 10% fetal bovine serum (FBS, Life Technologies, 13,140,071). For viral stock preparation, NCTC‐1469 or 17Cl‐1 cells were infected with stock MHV‐A59 or MHV‐JHM, respectively, in 175 cm^2^ tissue culture flasks at a multiplicity of infection (MOI) of 0.01 in 10 mL DMEM plus 2% heat inactivated horse serum (NCTC‐1469) or bovine serum (17Cl‐1) for 1 h at 37°C, then supplemented with an additional 8 mL of growth media and cultured at 37°C and 5% CO_2_ for 24 h. Infected cells were frozen in their flasks at −80°C for 24 h, then thawed in a water bath to release virus. Supernatant was centrifuged at 1300 ×g for 10 mins at 4°C. Viral supernatant was then collected and ultracentrifuged for 4 h at 110,500 xg in SW32Ti rotor. Virus pellet was resuspended in 1 mL TNE (10 mM TrisCl, 150 mM NaCl, 1 mM EDTA), then aliquoted and stored at −80°C until use. Viral titer was determined by plaque assay on L929 (CCL‐1) cells obtained from ATCC.

### 4.2 Mice and infections

Eight week old (8 week) C57BL/6 male mice were purchased from Charles River Laboratories and 18 month old (18 months) aged male mice were obtained from the National Institute on Aging Aged Rodent Colony, also housed at Charles River Laboratories. For MHV‐A59 infections, mice were deeply anesthetized with isoflurane, then infected intranasally (i.n.) with 10^3^ or 10^4^ pfu MHV‐A59 in 50 uL Hanks Balanced Salt Solution (HBSS, Life Technologies, 14,185,052) plus 1% fetal bovine serum (FBS, Life Technologies, 13,140,071). Mock infected controls received 50 μL inoculum of HBSS plus 1% FBS. For MHV‐JHM infections, mice were deeply anesthetized with isoflurane, then infected intracranially (i.c.) with 10 pfu MHV‐JHM in 10 uL HBSS +1% FBS. For adoptive transfer studies, 8 week male Ly5.1 (CD45.1, Charles River Laboratories) or 18 months C57BL/6 (CD45.2) animals were infected with 10^3^ pfu MHV‐A59 i.n., spleens harvested at 7 days post infection (DPI) and processed to single cell suspension. CD8^+^ T cells were purified using the CD8^+^ T cell negative selection isolation kit (Miltenyi Biotec, 130–104–075) according to manufacturer's instructions and diluted to 1 × 10^7^ cells/200 μL in PBS. CD8^+^ T cells were transferred intravenously (i.v.) via the tail vein in 200 μL into either 8 week Ly5.1 (CD45.1), 8 week C57BL/6 (CD45.2) or 18 months C57BL/6 mice (CD45.2), as indicated. All animal experiments were approved by the Institutional Animal Care and Use Committee of the University of North Carolina Charlotte.

### 4.3 Viral burden measurements

For detection of viral burden, mice were euthanized on designated DPI and blood collected via cardiac puncture into serum separator tubes (BD365967). After collection, tubes were centrifuged at 1300 rpm for 10 min at 4°C, and serum transferred to a new microcentrifuge tube. Animals were transcardially perfused with 20 mL cold 1X Phosphate Buffered Saline (PBS, Life Technologies, 14200166) and lungs, CLN, MdLN, and spleens collected. Brains were harvested and microdissected. All organs were snap frozen and weighed. Upon thawing, tissues were homogenized in 500 μL sterile PBS using a bead beater at 4 m/s for 1 min. Viral titers for tissues were assessed by plaque assay on L929 cells. Viral RNA in serum was determined by qRT‐PCR on RNA collected using E.Z.N.A. Viral RNA Kit (OMEGA Bio‐Tek, R6874‐01). MHV‐A59 N gene was quantified using a standard curve of known viral titer following qRT‐PCR with iTaq Universal SYBR Green One‐Step Kit (BioRad, 172–5151) and the following primers: Forward: 5’‐CAGATCCTTGATGATGGCGTAGT‐3′; Reverse: 5’‐AGAGTGTCCTATCCCGACTTTCTC‐3′ as previously reported (Yang et al., 2014).

### 4.4 Behavior testing

Anxiety and locomotor function was assessed using the Open Field assessment. A standard Open Field arena was used, which consisted of a square box (54 × 54 cm) with a grid (6 squares along each side) along the base. Animals were placed into the arena and allowed to explore for 5 min, at which point the animal was returned to its home cage and the arena sanitized with 70% ethanol between trials. Trials were recorded using Stoelting ANY‐maze USB 2 camera (Stoelting Co) and the number of lines crossed, total time spent in the center of the arena, and average speed was measured using ANY‐maze software. Spatial memory was assessed using an elevated Barnes Maze table (91.4 cm diameter, containing 19 empty holes and 1 target hole, spaced evenly 5 cm apart and 6.25 cm from the edge of the table). Mice were tested over 5 consecutive days, with 2 trials each day, exactly 30 min apart. Visual cues around the room were retained in the same location for the duration of the testing period. For each trial, the animal was placed into the center of the maze in a covered start box for 10 s, with removal of the box signaling start of the trial. Each animal was given 3 min to explore the maze and find the target hole. Animals that did not locate the target hole within 3 min were gently guided into it. After each trial, the animal remained in the target hole for exactly 1 min, then was returned to their home cage. The maze was decontaminated with 70% ethanol between trials. Trials were recorded using Stoelting ANY‐maze USB 2 camera and latency to find the target hole was determined using ANY‐maze software.

### 4.5 Tissue isolation for flow cytometry

Single cell suspension from tissues was performed as previously described (Shane et al., 2018). Briefly, after perfusion with 20 mL PBS, lungs were excised, minced and incubated for 30 min at 37°C with 1.25 mM EDTA, followed by 1 h incubation with 150 U/mL collagenase type IV (Life Technologies, 17–104‐019) diluted in RPMI (Life Technologies, 21–870–100) supplemented with 1.25 mM CaCl_2_ (Fisher, AC349610250), 1.25 mM MgCl_2_ (Fisher, AC223211000), 5% FBS, 500 mM HEPES (Life Technologies, 15630080), 200 mM L‐glut (Life Technologies, 21–051‐024), 2000 U/mL Antibiotic‐Antimycotic (Fisher, 15240062), and 5 ug/mL Gentamycin (Life Technologies, 15750060). Cells were then passed through a 40 μM cell strainer and resuspended in 44% isotonic Percoll (Fisher, 45–001–747) diluted in RPMI underlaid with 67% isotonic Percoll diluted in PBS, then centrifuged at 2800 rpm for 20 min at 4°C and the cellular interface collected. For isolation of leukocytes from the brain tissue, perfused brains were excised, minced and incubated for 1 h with 5 mg/mL collagenase type I (Sigma, C0130‐500MG) diluted in HBSS supplemented with 0.1 ug/mL TLCK trypsin inhibitor (Sigma, T754), 10 ug/mL DNase1 (Sigma, D4263‐0.5MG), and 10 mM HEPES. Cells were then passed through a 40 μM cell strainer and resuspended in 37% isotonic Percoll diluted in RPMI, centrifuged at 2800 rpm for 20 min at 4°C and cell pellet collected. Lymph nodes and spleens were mechanically digested by passage through a 40 μM cell strainer and collected in RPMI. Erythrocytes were removed from spleens using ACK lysis buffer (Life Technologies, A1049201). Live cell numbers of all samples were determined by staining with Trypan blue (Life Technologies, 15250061) and counted using Bio‐Rad TC‐20 Automated Cell Counter.

### 4.6 Flow cytometry staining

Prior to immunostaining, all cells were blocked with 1:50 dilution of TruStain FcX anti‐mouse CD16/CD32 (Clone 93, Biolegend) and Fixable Viability Dye (eBioscience, 65–0866‐18) for 5 mins. The immunodominant MHV spike protein (S) MHC class I tetramer [H‐2K^b^/RCQIFANI] was generated at the National Institute of Allergy and Infectious Diseases Tetramer Facility, as described previously (Cupovic et al., 2016). Tetramer staining was carried out at room temperature for 20 min in conjugation with other surface staining antibodies: CD8a (53–6.7, APC‐Cy7), CD4 (RM4‐5, APC), CD44 (IM7, PE‐Cy7), CD45 (30‐F11, APC), CD45.1 (20–0453, APC), CD45.2 (60–0454, PE‐Cy‐7), CD11b (M1/70, FITC), P2RY12 (S16007D, PE), CD68 (FA‐11, BV‐421), MHC II (M5/114.15.2, PerCP‐Cy5.5), obtained from Cytek Biosciences or eBioscience, then washed with 1× PBS and fixed with 2% paraformaldehyde (PFA). For assessment of intracellular cytokine production, cells were harvested as described above and treated with eBioscience Cell Stimulation Cocktail (00–4970–03) and eBioscience Protein Transport Inhibitor (00–4980‐03) for 5 h at 37°C. Following stimulation, cells were stained for cell surface markers, then permeabilized using eBioscience PermWash and intracellularly stained as per the manufacturer instructions (00–5523‐00) for IFN‐γ (XMG1.2, APC), TNF (MP6‐XT22, PerCP‐710), perforin (eBio0MAK‐D, FITC), and granzyme B (NGZB, PE‐Cy7). Cells were then fixed in 2% paraformaldehyde for flow cytometry analysis.

### 4.7 Flow cytometry analysis

Flow cytometry data were acquired using a BD Fortessa with FACSDiva Software (BD Biosciences) and analyzed using FlowJo software version 10.9.0 (BD Biosciences). As shown in Figure S2a, lymphocytes were gated as follows: single cells (FSC‐A × FSC‐H), live (viability dye negative), lymphocytes (FSC‐A × SSC‐H; lymphocyte subpopulation only), then stained with cell‐specific antibodies, as indicated in figure legends. For microglial assessment samples were gated as follows: single cells (FSC‐A × FSC‐H), live (viability dye negative), leukocytes (FSC‐A × SSC‐H; lymphocyte + granulocyte/myeloid cells), then stained with cell‐specific antibodies, as indicated in figure legends.

### 4.8 Immunohistochemistry

At designated timepoints post infection, animals were euthanized and perfused with 20 mL cold PBS, then brains were excised and fixed overnight in 4% PFA. Following fixation brains were cryoprotected in two exchanges of 30% sucrose diluted in PBS, then embedded and frozen in OCT (Fisher, 23–730–571). Sagittal sections were cut to 10 μm using a Microm HM550 cryostat. Prior to immunostaining, sections were blocked for 1 h. at room temperature with 5% goat serum/0.1% Tween‐20/PBS, then stained at 4°C overnight with combinations of the following antibodies: rat anti‐CD8a (1:1000, eBioscience 53–6.7), rabbit anti‐NeuN (1:1000, Cell Signaling Technologies, 12943S), rabbit anti‐Iba1 (1:1000, Wako Chemicals, 019–19741), rabbit anti‐GFAP (1:1000, Cell Signaling Technologies, 12389S), guinea pig anti‐synaptophysin (1:500, Cedar Lane Labs, 101004[SY]), guinea pig anti‐doublecortin (1:500, MilliporeSigma, AB2253), mouse anti‐MHV Nucleocapsid (1:1000, BEI, NR‐45106). Sections were then washed with 0.1% Tween‐20/PBS and incubated for 1 h at room temperature with the following fluorescently labeled secondary antibodies: Alexa Fluor 647 anti‐Rabbit (1:400, Life Technologies, A‐21245), Alexa Fluor 488 anti‐Guinea‐Pig (1:400, Life Technologies, A‐11073), Alexa Fluor 488 anti‐Rat (1:400, Life Technologies, A‐11006). Sections were then counterstained with 1 μg/mL DAPI and coverslipped with Prolong Gold Antifade Mountant (Fisher, P36930). Samples were treated with TUNEL staining reagent as prepared by manufacturer's instructions (Sigma‐Aldrich, 12–156–792–910) prior to blocking and primary staining. Images were captured using a Keyence BZX‐800 at 20× and 40× objective magnification. Images were processed and quantified using Fiji ImageJ (Schindelin et al., 2009).

### 4.9 In vitro CD8^+^ T cell neuron cocultures

Primary cortical neurons were isolated from embryos collected from timed‐pregnant C57BL/6 mice on embryonic day 18 as previously described (Funk & Lotz, 2020). Briefly, cerebral cortices were dissected from embryonic brains, treated with papain (20 U/mL) and DNase 1 (2.5 U/mL) for 30 min, and gently dissociated by trituration in Hibernate E medium (Life Technologies, A1247601). Neurons were diluted in 5 mL Neuron Growth Medium (Neurobasal media (Life Technologies, A3582901) supplemented with 2% B27 (Life Technologies, 17504001), 2 mM L‐glut (Life Technologies, 35050–061) and 100 U/mL Antibiotic‐Antimycotic (Fisher, 15240062)) at a concentration of 1 × 10^5^ neurons/ml so that 1 × 10^5^ neurons were seeded onto poly‐D‐lysine (10 μg/mL) coated acid‐etched coverslips (VWR, MSPP‐P06G1520F) and cultured at 37°C plus 5% CO_2_. Half‐media changes were performed every 72 h until neurons were used for experiments at 7–10 days in vitro, as indicated in text.

CD8^+^ T cells were isolated from the spleens of uninfected or MHV‐A59 infected animals using the CD8^+^ T cell negative isolation kit (Miltenyi Biotec, 130–104–075) according to manufacturer's instructions. CD8^+^ T cells were seeded onto neurons at a concentration of 1 × 10^5^ cells/well (1:1 ratio) for 24 h, at which point culture supernatant was collected and cryopreserved, and coverslips were co‐stained for TUNEL and NeuN, as described above. Total NeuN^+^ or TUNEL^+^NeuN^+^ cells were quantified using the ImageJ Cell Counter plugin.

### 4.10 Statistics

Statistical analyses were performed using Graph Pad Prism software version 9.3.1 or 10.0.2.

## AUTHOR CONTRIBUTIONS

KLR, RL, and KEF contributed to experimental design and data analyses. KLR, RL, LAW, and LC performed experiments and generated figures. KLR and KEF contributed to the writing and editing of the manuscript. All authors have reviewed the manuscript for accuracy and agree with the final content as presented.

## CONFLICT OF INTEREST STATEMENT

The authors declare no conflict of interest.

## Supporting information


Appendix S1.

## Data Availability

All data needed to evaluate the conclusions of this study are presented in this article and its supporting information. All data analyzed or generated during the study are available from the corresponding author upon reasonable request.
