# Supplementary material for: Compromised CD8+ T cell immunity in the aged brain increases severity of neurotropic coronavirus infection and postinfectious cognitive impairment
Source: Aging Cell. 2024 Nov 17;24(3):e14409. doi: 10.1111/acel.14409 (PMC11896202; doi:10.1111/acel.14409)
Supplement: Supplementary file 1 — Appendix S1. [file ACEL-24-e14409-s001.docx]

**Supporting Information**


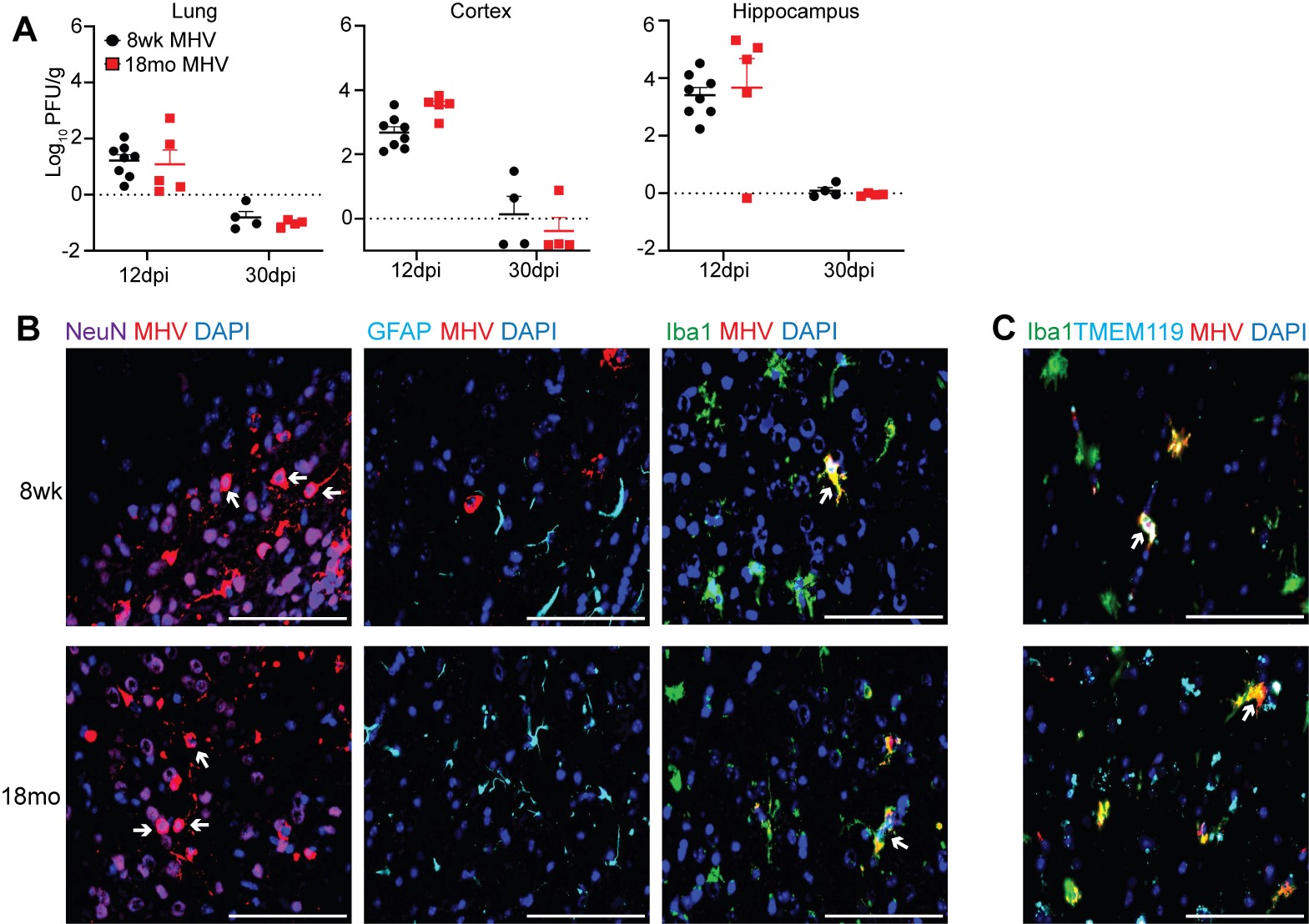


**Supplemental Figure 1.** **Assessment of MHV-A59 CNS infection**

(**A**) Quantification of MHV genome (N gene) by qRT-PCR. (**B**) Representative IHC of MHV N protein (red) co-stained with NeuN (purple), GFAP (cyan), or Iba1 (green) in adult or aged brains at 6 DPI. NeuN^+^MHV^+^ and Iba1^+^MHV^+^ cells indicated by white arrows. (**C**) Representative IHC of MHV-N protein (red) co-stained with Iba1 (green) and TMEM119 (cyan) in adult or aged brains at 6 DPI. Iba1^+^TMEM119^+^MHV^+^ cells indicated by white arrows. Images acquired at 40X magnification. Scale bar = 100 µm. No statistical significance at p<0.05 was detected according to two-way ANOVA.


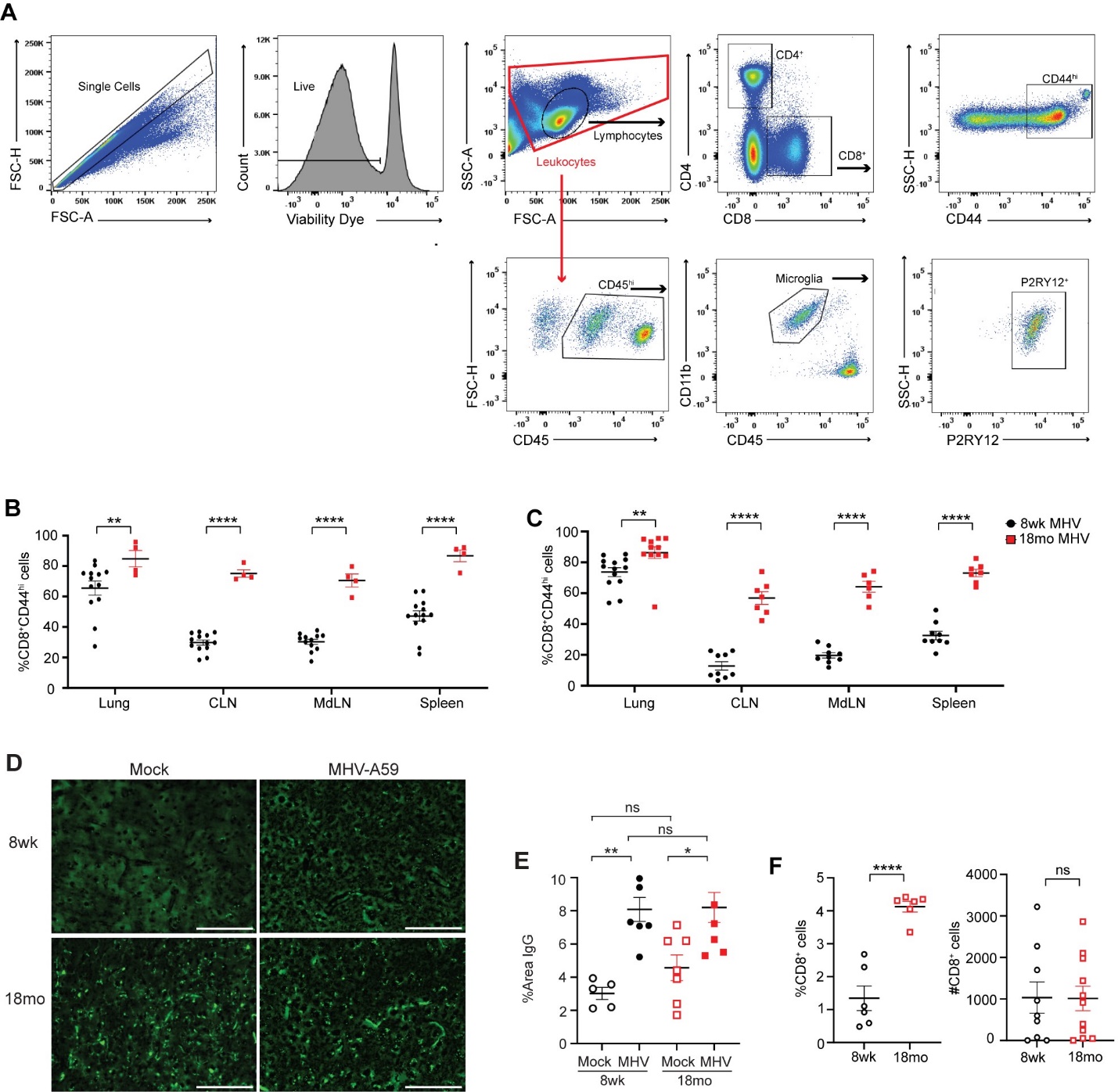


**Supplemental Figure 2**. **Enhanced CD8^+^ T cells responses in peripheral compartments of aged mice following MHV-A59 infection.**

(**A**) Representative flow cytometry gating strategy of lymphocytes and leukocytes for assessment of CD8^+^ T cells and microglia. 8 wk adult or 18 mo aged C57BL/6 mice were infected with (**B**) 10^4^ pfu MHV-A59 or (**C**) 10^3^ pfu MHV-A59, and frequency of lymphocytes expressing CD8^+^CD44^hi^ at 30 DPI in peripheral tissues were quantified by flow cytometry. (**D**) Representative IHC staining for IgG in the cortex of 8 wk adult or 18 mo, mock or MHV-A59 infected animals. Images taken at 40X magnification. Scale bar = 100 µm. (**E**) Quantification of total percent area of IgG in the cortex. (**F**) 8 wk adult or 18 mo aged C57BL/6 mice were mock-infected with HBSS + FBS i.n., then at 30 days post-inoculation the percentage of lymphocytes that were CD8^+^ as well as the total number of CD8^+^ T cells in the brains were quantified by flow cytometry. Data representative of 2-3 independent experiments with each data point representing a single animal. Statistics according one-way-ANOVA. *, p<0.05; **, p<0.01; ***, p<0.001; ****, p<0.0001.


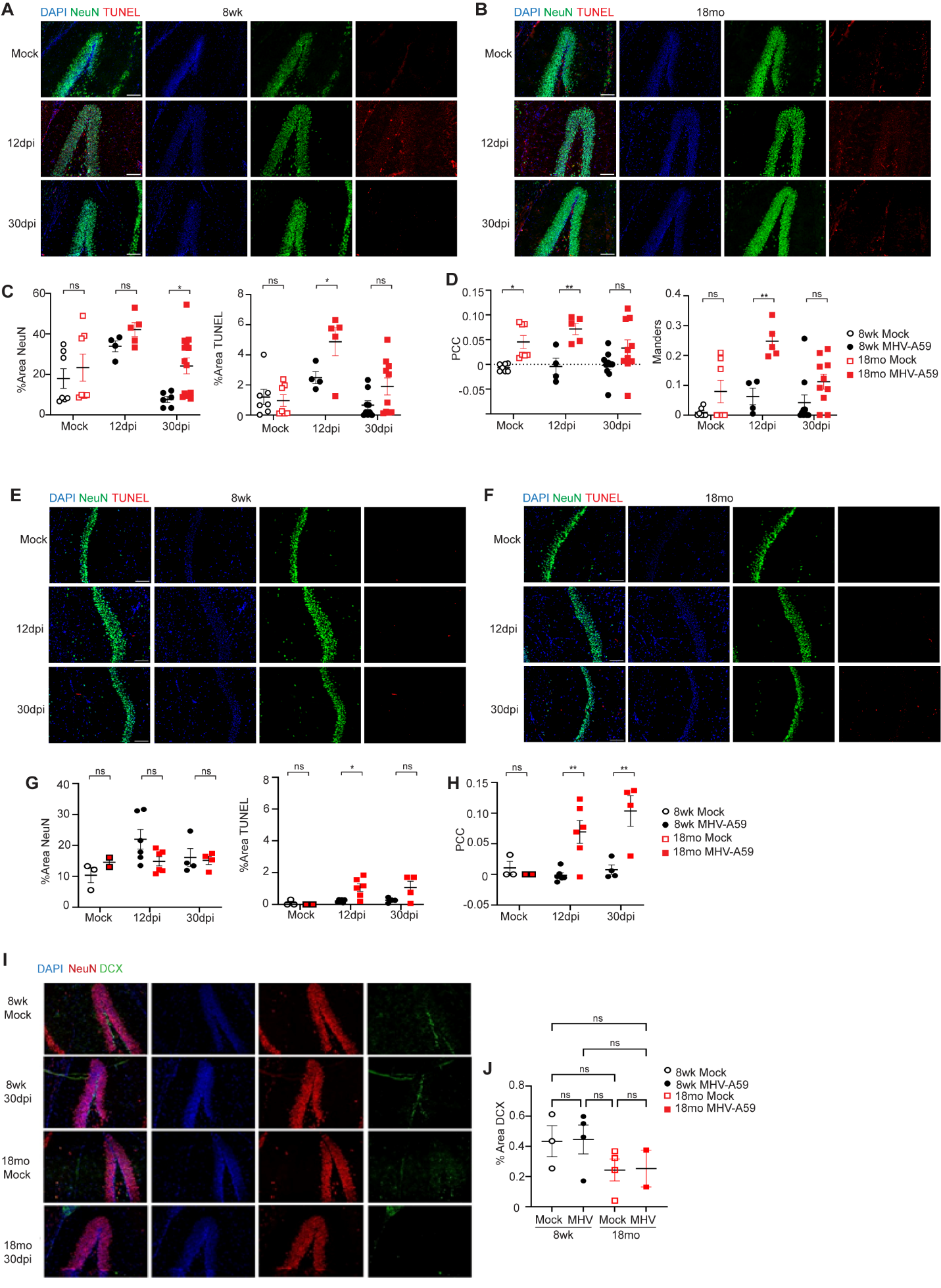


**Supplemental Figure 3.** **Aged animals demonstrate enhanced neuronal apoptosis and impaired neuronal regeneration following MHV-A59 primary infection.**

Representative IHC of DAPI, NeuN, and TUNEL in the DG of (**A**) 8 wk or (**B**) 18 mo old animals at 12 DPI and 30 DPI compared to mock infected controls. Images taken at 20X magnification. Scale bar = 100 µm. (**C**) Quantification of total percent area of NeuN and TUNEL in the DG. (**D**) Colocalization between NeuN and TUNEL measured by PCC and Mander’s overlap coefficient. Representative IHC of DAPI, NeuN, and TUNEL in the CA1 of (**E**) 8 wk or (**F**) 18 mo old animals at 12 DPI and 30 DPI compared to mock infected controls. Images taken at 20X magnification. Scale bar = 100 µm. (**G**) Quantification of total percent area of NeuN and TUNEL in the CA1. (**H**) Colocalization between NeuN and TUNEL measured by PCC. Data representative of 2 experiments with each data point representing a single animal. **(I)** Representative IHC of DAPI, NeuN, and DCX in the DG of 8 wk and 18 mo mock- or MHV-A59 infected animals at 30 DPI. (**J**) Quantification of total percent area of DCX in the DG. Data representative of 1 experiment with each data point representing a single animal. Statistics according to unpaired one-way ANOVA. *, p<0.05; **, p<0.01; ***, p<0.001; ****, p<0.0001.


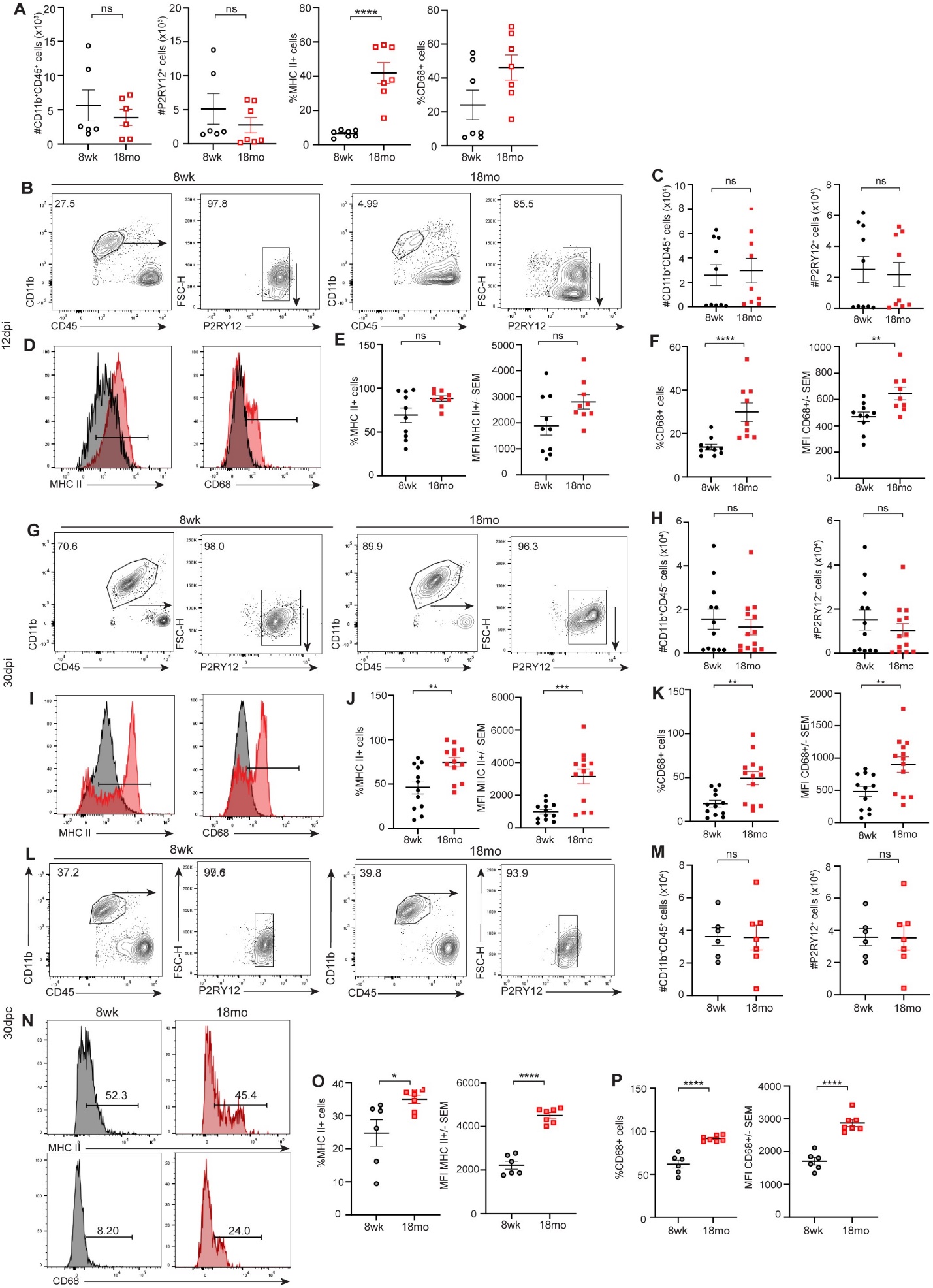


**Supplemental Figure 4.** **Assessment of microglia responses in adult vs aged animals at steady state and following MHV-A59 infection**

(**A**) Mock-infected 8 wk adult or 18 mo aged C57BL/6 mice were assessed for total number of myeloid cells (CD11b^+^CD45^+^) and microglia (P2RY12^+^CD11b^+^CD45^+^), as well as the proportion of microglia (P2RY12^+^CD11b^+^CD45^+^) positive for MHC II^+^ and CD68^+^. 8 wk adult or 18 mo aged C57BL/6 mice were infected with 10^3^ pfu MHV-A59 and microglial numbers and activation were assessed at (**B-F**) 12 DPI and (**G-K**) 30 DPI. **(B, G)** Representative flow cytometry of CD11b^+^CD45^+^ myeloid cells and CD11b^+^CD45^+^P2RY12^+^ microglia 12 and 30 DPI. (**C, H**) Quantification of total number of myeloid cells and microglia. (**D, I**) Representative histograms of MHC II and CD68 expression by microglia (P2RY12^+^CD11b^+^CD45^+^) at 12 and 30 DPI. (**E, J**) Quantification of frequency and MFI of MHC II^+^ on microglia. (**F, K**) Quantification of frequency and MFI of CD68^+^ on microglia . (**L**) Representative flow cytometry of CD11b^+^CD45^+^ myeloid cells and CD11b^+^CD45^+^P2RY12^+^ microglia 30 DPC (as described in Figure 4). (**M**) Quantification of total number of myeloid cells and microglia. (**N**) Representative histograms of MHC II and CD68 expression by microglia (CD11b^+^CD45^+^P2RY12^+^) at 30 DPC. Quantification of frequency and MFI of (**O**) MHC II^+^ and (**P**) CD68^+^ expression on microglia . Data were pooled from 2-3 independent experiments with each data point representing a single animal. Statistics according to unpaired student t-test. *, p<0.05; **, p<0.01; ***, p<0.001; ****, p<0.0001.


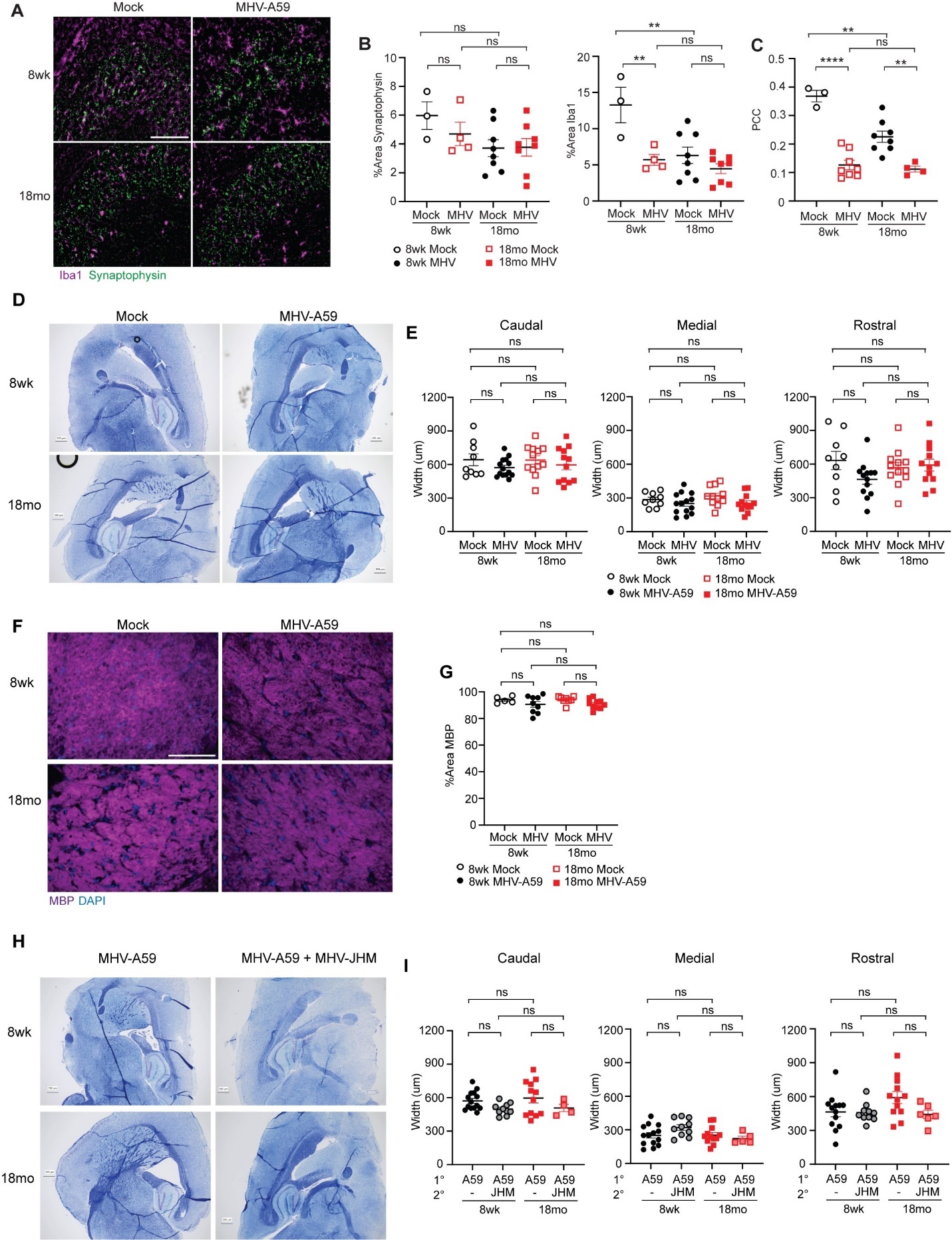


**Supplemental Figure 5.** **Assessment of microglia mediated synaptic elimination and demyelination following primary MHV-A59 infection.**

(**A**) Representative IHC images of Iba1 and Synaptophysin in the CA3 region of the hippocampus of 8 wk or 18 mo old animals at 30 DPI. Images taken at 40X magnification. Scale bar = 100 µm. (**B**) Quantification of percent area of Synaptophysin and Iba1 staining. (**C**) Quantification of PCC for synaptophysin and Iba1 colocalization. (**D)** Representative brightfield images of Luxol Fast Blue staining in the corpus callosum of 8 wk or 18 mo old MHV-A59 or mock infected animals 30 DPI. Images taken at 2X magnification. Scale bar = 500 µm. (**E**) Quantification of caudal, medial, and rostral corpus callosum width. (**F**) Representative IHC images of myelin basic protein (MBP) and DAPI staining within the corpus callosum of 8 wk or 18 mo old MHV-A59 or mock infected animals 30 DPI. Images taken at 40X magnification. Scale bar = 100 µm. (**G**) Quantification of percent area of MBP in the corpus callosum. Images taken at 40X magnification. Scale bar = 100 µm. (**H)** Representative brightfield images of Luxol Fast Blue staining in the corpus callosum of 8 wk or 18 mo old mice infected with MHV-A59 alone or MHV-A59, then MHV-JHM at 30 DPI/DPC. Images taken at 2X magnification. Scale bar = 500 µm. (**I**) Quantification of caudal, medial, and rostral corpus callosum width. Statistics according to unpaired one-way ANOVA. *, p<0.05


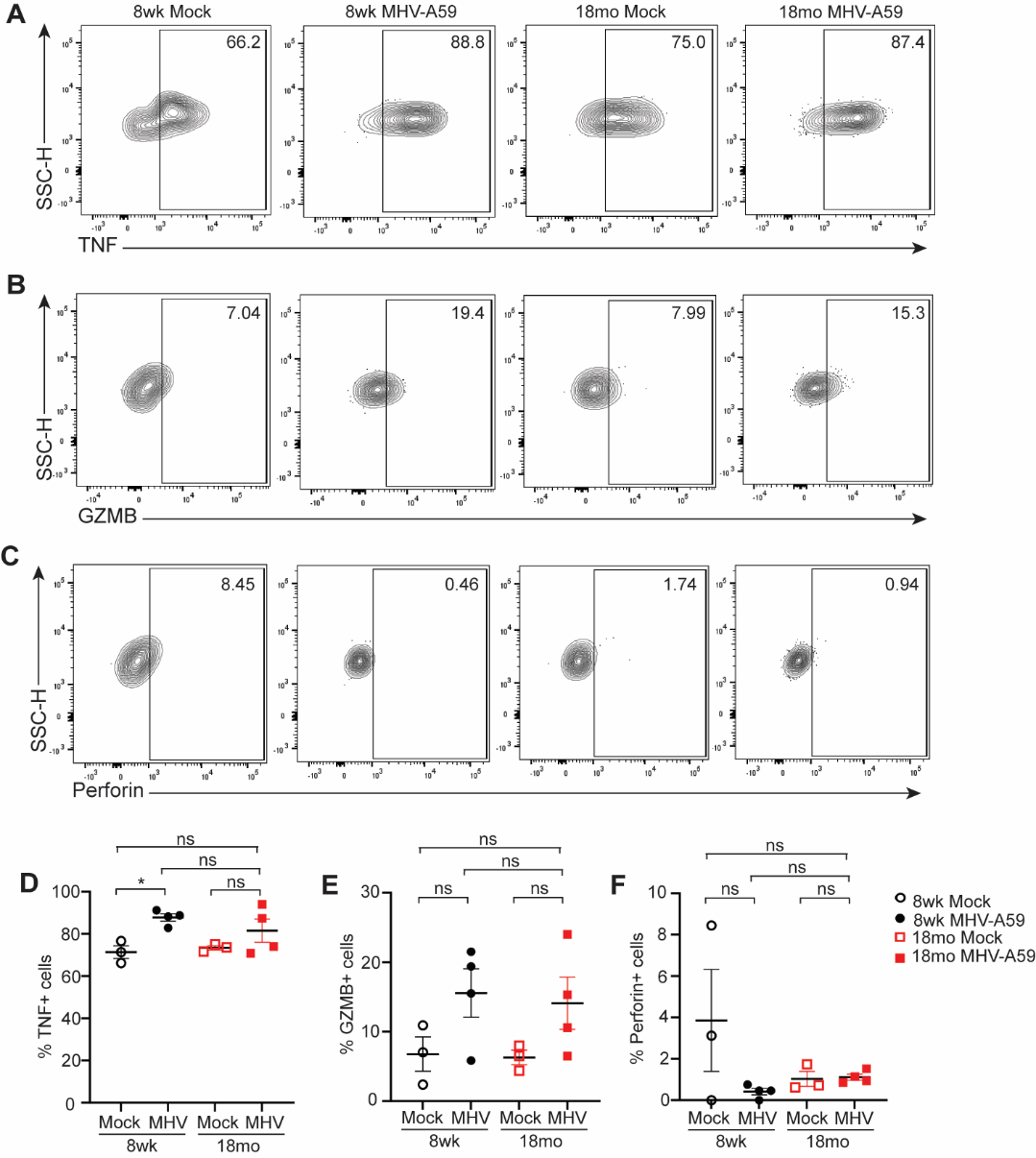


**Supplemental Figure 6.** **Production of TNF, GZMB, and Perforin by 8 wk or 18 mo old animals at 30 DPI**

Representative flow cytometry plots of (**A**) TNF^+^, (**B**) GZMB^+^, or (**C**) Perforin^+^ on CD8^+^CD44^+^ T cells isolated from the brain of 8wk or 18month old MHV-A59 or mock infected animals 30 DPI following 4 hr PMA/ionomycin stimulation. Quantification of percent frequency of CD8^+^ CD44^+^ lymphocytes expressing (**D**) TNF, (**E**) GZMB, or (**F**) Perforin. Cells previously gated on live, single cell, CD8^+^ CD44^+^ lymphocytes. Statistics according to unpaired one-way ANOVA. *, p<0.05
